# Solid‐Phase‐Supported Chemoenzymatic Synthesis of a Light‐Activatable tRNA Derivative

**DOI:** 10.1002/anie.202111613

**Published:** 2021-11-22

**Authors:** Anja Blümler, Harald Schwalbe, Alexander Heckel

**Affiliations:** ^1^ Institute for Organic Chemistry and Chemical Biology Goethe University Frankfurt am Main Max-von-Laue-Strasse 7 60438 Frankfurt/Main Germany; ^2^ Institute for Organic Chemistry and Chemical Biology Center for Biomolecular Magnetic Resonance BMRZ Goethe University Frankfurt am Main Max-von-Laue-Strasse 7 60438 Frankfurt/Main Germany

**Keywords:** chemoenzymatic synthesis, ligases, light control, RNA synthesis, solid-phase synthesis

## Abstract

Herein, we present a multi‐cycle chemoenzymatic synthesis of modified RNA with simplified solid‐phase handling to overcome size limitations of RNA synthesis. It combines the advantages of classical chemical solid‐phase synthesis and enzymatic synthesis using magnetic streptavidin beads and biotinylated RNA. Successful introduction of light‐controllable RNA nucleotides into the tRNA^Met^ sequence was confirmed by gel electrophoresis and mass spectrometry. The methods tolerate modifications in the RNA phosphodiester backbone and allow introductions of photocaged and photoswitchable nucleotides as well as photocleavable strand breaks and fluorophores.

## Graphical abstract

Synthetic access to modified RNA is of high importance. Both the gold standard—chemical solid‐phase synthesis—and enzymatic methods have limitations. The present study offers a best‐of‐both‐worlds approach and enabled the synthesis of a tRNA derivative with four modifications. This approach is compatible with a whole toolbox of light‐reactive and other modifications.

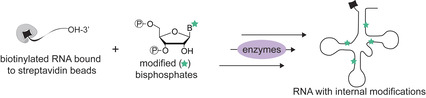

RNA plays an essential role in many biological processes. In order to understand the underlying intricate mechanisms and to learn about the structural and dynamic aspects especially of regulatory RNA, there is increasing demand for reliable methods to synthesize modified RNA with novel functions, different stability and a variety of biophysical probes.

The traditional automated chemical solid‐phase synthesis^[1]^ is a powerful technique. It allows introduction of an arbitrary number of modifications into oligonucleotides in an efficient and position‐specific manner. There is, however, a limit to the possible RNA oligonucleotide length. Depending on the modification and the sequence, the synthesis and isolation of highly pure modified RNAs longer than 50 nucleotides remain a challenge.[^2^, ^3^, ^4^, ^5^, ^6^] Reports of longer sequences of modified RNA, prepared by solid‐phase methods, exist, but the experimental struggle grows fast with length and complicatedness of the modification.[^6^, ^7^]

On the other hand, there are the enzymatic approaches based on in vitro transcription using *T7* RNA polymerases[^8^, ^9^, ^10^, ^11^] or modifications using transferases.[^12^, ^13^, ^14^, ^15^, ^16^, ^17^] Enzymatic methods allow synthesis essentially without size limitation; preparation of RNAs comprising 400 nt has been reported.[^18^, ^19^] However, severe limitations exist for the preparation by template‐driven synthesis with *T7* polymerase. Watson–Crick base‐pairing between the DNA template strand and the incoming nucleotide determines which of four RNA triphosphates is incorporated. Thus, position‐specific labeling is difficult.[^19^, ^20^, ^21^, ^22^]

Chemoenzymatic approaches can be a solution: we reported earlier that a combination of two different *T4* RNA ligases and modified 5′,3′‐bisphosphates enabled us to synthesize a 392mer RNA modified at one specific internal position.^[23]^ This approach does not use the harsh conditions of chemical solid‐phase synthesis and thus allows the introduction of more delicate nucleotide modifications.

In the present study, we apply the best of both worlds and combine the advantages of solid‐phase synthesis and chemoenzymatic methods to arrive at the introduction of multiple modifications at specific positions of an RNA. As test case, we synthesize a derivative of the tRNA coding for methionine (Scheme 1 a).^[24]^ With a length of 70–90 nucleotides, a normal solid‐phase synthesis of tRNAs is very difficult, especially if several modifications have to be introduced.^[25]^

**Scheme 1:**
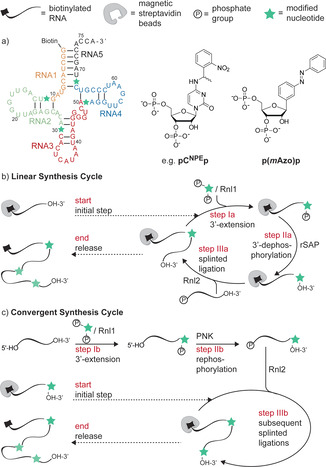
a) Sequence of the tRNA derivatives synthesized in this study (see the Supporting Information for the structure of **pG^NPE^p** and **pU^NPE^p**). b) Linear chemoenzymatic synthesis cycle. c) Convergent approach including 3′‐extension in solution. NPE: 1‐(2‐nitrophenyl)ethyl.

Light‐responsive modifications find numerous applications in oligonucleotide research.[^7^, ^26^, ^27^, ^28^, ^29^] Recent examples investigated RNA folding dynamics,[^30^, ^31^] regulation of biological processes[^7^, ^32^, ^33^] or labeling strategies.[^34^, ^35^, ^36^] “Photocages”—such as the photolabile 1‐(2‐nitrophenyl)ethyl (NPE) group—temporarily influence or even block the structural or functional behavior of biomolecules. Irradiation with light at a certain wavelength leads to cleavage of the protecting group and irreversible recovery of the molecule's native form and function.[^37^, ^38^] Photoswitches, such as azobenzene, offer the possibility of reversible regulation.[^39^, ^40^, ^41^, ^42^] Both types of light‐controllable compounds thus enable non‐invasive highly specific spatiotemporal control.[^43^, ^44^, ^45^]

Our chemoenzymatic method consists of three enzymatic steps. In the first step, an existing RNA is 3′‐extended with a nucleoside 5′,3′‐bisphosphate using *T4* RNA ligase 1 (Rnl1).^[46]^ In this step, the 3′‐phosphate serves as protecting group against multiple 3′‐extensions. It is removed in the second step using shrimp alkaline phosphatase (rSAP). The third step is then a splinted ligation with a second RNA fragment using *T4* RNA ligase 2 (Rnl2) and a subsequent digestion of the DNA splint with DNase.^[47]^

In this study, we establish the repeated application of this procedure (Scheme 1) and applied a solid‐phase strategy. As support we used magnetic streptavidin beads,^[48]^ which can interact with 5′‐biotinylated RNA. This 5′‐handle can be optionally removed at the end of the synthesis (vide infra).

For the modification, a set of nucleotide bisphosphates (**pC^NPE^p**, **pG^NPE^p** and **pU^NPE^p** or the bisphosphate **p(*m*Azo)p** of a *meta*‐substituted azobenzene *C*‐nucleoside analogue (*m*Azo)) was used.^[23]^ Four positions in the tRNA sequence were chosen for the modification—one in each stem of its cloverleaf structure. This decision for labeling positions breaks the synthesis down to five unmodified RNA fragments (**RNA1**–**RNA5**) with lengths that can be readily prepared using solid‐phase synthesis and purified by HPLC.

**RNA1** (orange sequence in Scheme 1 a) with a 5′‐biotin was bound to streptavidin beads. This sequence was 3′‐extended with either **pC^NPE^p** or **p(*m*Azo)p** using the enzyme Rnl1. Careful optimization of the conditions led to an increase in yield from 70 % to 93 % in both cases. Interestingly, the key changes were a reduction in the excess of bisphosphate (from 1:4 to 1:3) as well as a reduction in reaction time (from an overnight reaction to 3–8 h). For comparison, the reaction was also performed in solution (Figure 1 a) with no difference in yield (see Supporting Information Figure S3). For the dephosphorylation step, the buffer was exchanged. This buffer exchange is greatly facilitated by the bead‐supported RNA. Dephosphorylation was performed using rSAP and was quantitative both in solution and on solid support (see Supporting Information Figure S6). After a second buffer exchange, **RNA2** (green sequence in Scheme 1 a) was ligated using a DNA splint and Rnl2. Ligations of RNA strands are known to be notoriously difficult. In this case, after careful optimization, we could achieve 82 % yield at 37 °C for this step both for the incorporation of **C^NPE^** and ***m*** **Azo** (76 % total yield up to that point in a batch size of for example 0.8 nmol, see Figure 1 c). A relative ratio of **RNA1**:**RNA2**:**splint**=1:1:1 turned out to be optimal. Also, DNA ligase buffer afforded higher yields than the original buffer recommended for Rnl2. Again, there was no noticeable difference between performing the reaction on solid support or in solution (see Supporting Information Figure S9).

**Figure 1:**
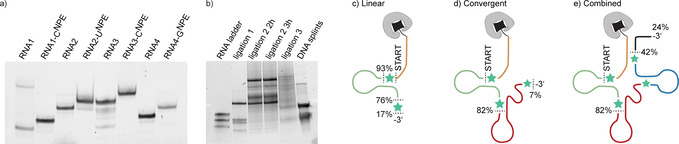
a) Polyacrylamide gel analysis of the commercially obtained RNA building blocks **RNA1**–**RNA4** and their 3′‐extended products obtained in solution and after HPLC‐purification. b) Polyacrylamide gel analysis of ligations on the solid support. Ligation 1: **RNA1‐C^NPE^** +**p‐RNA2‐U^NPE^**, ligation 2: **RNA1‐C^NPE^‐RNA2‐U^NPE^** +**p‐RNA3‐C^NPE^‐RNA4‐G^NPE^**, ligation 3: **RNA1‐C^NPE^‐RNA2‐U^NPE^‐RNA3‐C^NPE^‐RNA4‐G^NPE^** +**p‐RNA5**. c–e) Total yields in the three synthetic strategies explored in this study. The values given are for the synthesis with incorporated NPE‐modified nucleosides. The yields for the synthesis with **mAzo** *C*‐nucleosides were similar (see text).

However, the significant handling advantage of the solid‐phase‐supported procedure was apparent, because in solution the workup of every step required either precipitation or RP‐HPLC, while the solid‐phase route required only washing. Importantly, if a purification step was required after one of the operations on solid support or if the beads degraded during the repeated operations, our particular choice of immobilization technique would allow releasing the sequence from the beads, carrying out a purification step and reattaching the sequence to new beads to continue the synthesis.

For the next cycle, the 29mer sequence (**RNA1‐C^NPE^‐RNA2**) was 3′‐extended with either **pU^NPE^p** or **p(*m*Azo)p** again using Rnl1. After optimization, we only obtained total yields (over the entire synthesis up to that point) of up to 17 % and 22 % for **RNA1‐C^NPE^‐RNA2‐U^NPE^** and **RNA1‐*m*Azo‐RNA2‐*m*Azo**, respectively (see Figure 1 c). Also, in the HPLC analysis after a test cleavage we found that it was difficult to separate the product from the unreacted 29mer by RP‐HPLC. While in chemical solid‐phase synthesis, the terminal DMTr group of a full‐length product typically affords significant shifts in an RP‐HPLC and thus allows separation even from N‐1 sequences, this is not possible in the chemoenzymatic method.

Therefore, we also developed a convergent strategy (Scheme 1 c) in which the respective new RNA stretches are 3′‐pre‐extended in solution, purified and only then ligated on the solid phase. In solution, **RNA2** could be 3′‐extended with yields of 87 % and 82 % for **pU^NPE^p** and **p(*m*Azo)p** for this single step, respectively (Figure 1 a). Here, the 3′‐extended product is significantly more lipophilic than the unextended one and could be easily purified by RP‐HPLC. The retention on RP‐HPLC differs by up to 3 min. The next two steps could be combined in one, as *T4* polynucleotide kinase can simultaneously 3′‐dephosphorylate and 5′‐phosphorylate the fragment quantitatively. The extended fragments of **RNA2**, prepared in solution and purified, were used to perform the splinted ligation (“ligation 1” in Figure 1 b) on solid support containing either **RNA1‐C^NPE^** or **RNA1**‐***m*** **Azo**. Using this convergent strategy, we obtained a total yield of 82 % for the 30mer with either two NPE‐modified residues or two ***m*** **Azo** residues (Figure 1 d).

For the third synthesis cycle, we combined the strength of the chemical and enzymatic synthesis methods and prepared the two 37mer fragments **RNA3‐C^NPE^‐RNA4** and **RNA3‐*m*Azo‐RNA4** by chemical solid‐phase synthesis (red‐blue fragment in Figure 1 e). After RP‐HPLC purification, the fragments were 3′‐extended with **pG^NPE^p** and **p(*m*Azo)p** in solution. Yields for this step were 69 % and 78 %, respectively. These building blocks, prepared in solution, could be ligated to their respective upstream 30mer fragments on solid support with yields of 51 % for this step in both cases (“ligation 2” in Figure 1 b). Thus, an overall yield of 42 % and 39 % was obtained for the 68mer with either four NPE‐modified nucleotides or four **mAzo** residues, respectively (Figure 1 e).

We had also tried to pre‐extend **RNA3** in solution using either **pC^NPE^p** or **p(*m*Azo)p**. This was possible with optimized yields of 42 % and 36 % for this step, respectively (Figure 1 a). However, the subsequent ligation to the previously prepared 30mers was very inefficient (Figure 1 d) even after optimization.

The final step in order to arrive at the fourfold modified 77mer consisted of a solid‐phase ligation of an unmodified 9mer and could be performed with a yield of 57 % and 49 % for this step, respectively. The final 77mer products were released from the streptavidin beads by heating to 75 °C and were obtained in a total yield of 24 % for the NPE‐modified tRNA and 19 % for the **mAzo**‐modified tRNA (Figure 1 e).

Thus, the convergent synthesis approach, allowing for purification at any required step after successive 3′‐extensions, enables the controlled high yield synthesis of modified tRNAs. Figure 2 shows the assigned RP‐HPLC chromatogram of the final products with the NPE modifications along with LC–MS data for the respective main peaks. While the LC–MS results of the intermediate fragments are very nice, it is clearly visible that also LC–ESI‐MS characterization approaches its limits with a modified 77mer.

**Figure 2:**
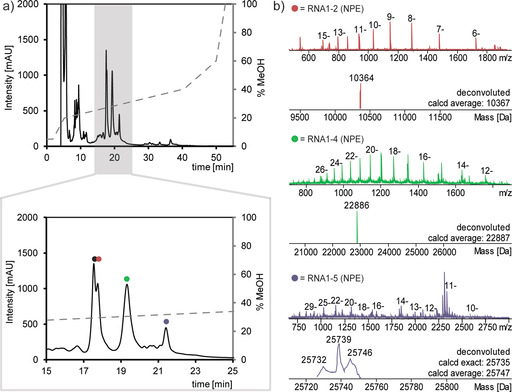
a) RP‐HPLC chromatogram of the final purification after solid‐phase‐supported synthesis of the tRNA containing four NPE‐caged nucleotides and zoomed area of interest. b) Mass spectra of the identified RNA fragments (•=**RNA1** (for mass spectra see Supporting Information Section 6.2), •=product of “ligation 1”, •=product of “ligation 2”, •=product “ligation 3”, for the explanation of “ligation *n*” see the caption of Figure 1).

After probing the repetition of the synthesis cycle and introduction of a solid support, we tested the scope of this method regarding the compatibility with different photoactivatable groups. This allows for example, for wavelength‐controlled sequential recovery of base‐pairing (Figure 3).^[49]^ **pU^DEACM^p** (Figure 3 a), a 5′,3′‐bisphosphate of a uridine nucleobase‐caged with a coumarin‐based DEACM photolabile protecting group could be used for the 3′‐extension of **RNA2** and subsequent solid‐phase‐supported ligation to **RNA1‐C^NPE^** (Figure 3 a). We could show the sequential uncaging by irradiation at 455 nm and 365 nm.

**Figure 3:**
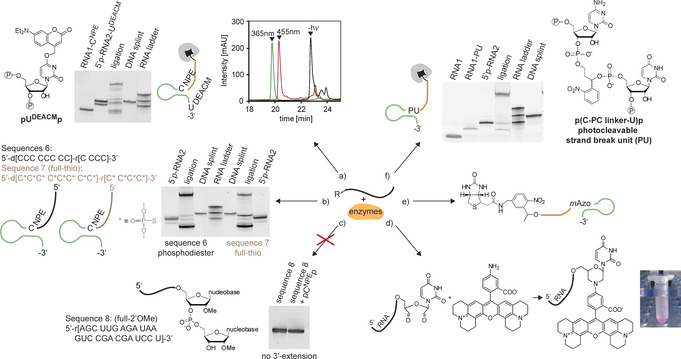
Overview of modification options explored in this study. a) Incorporation of NPE and DEACM photocaging groups for wavelength‐selective uncaging experiments. An overlay of HPLC traces before and after sequential uncaging is shown. b) 3′‐Extension and splinted ligation of DNA/RNA chimera with either a phosphodiester or a phoshorothioate (*) backbone. c) 3′‐Extension of 2′‐OMe RNA was unfortunately not possible. d) NaIO_4_‐capping of the 3′‐end of unmodified RNA allows a following rhodamine modification using morpholino chemistry. e) Introduction of a photocleavable linker enables light‐induced cleavage of the 5′‐biotin tag after the incorporation of *m*Azo photoswitches into RNA. f) Enzymatic incorporation of an internal photocleavable strand break unit that could be used for light‐induced backbone cleavage at defined positions.

Phosphorothioate chemistry is a well‐established method to confer stability against nucleolytic digestion. Figure 3 b shows that DNA/RNA mixmers and phosphorothioates can be prepared (sequence 6 in Figure 3 b and sequence 7 in Figure 3 b, respectively). However, an attempt to do the same with an exemplary 2′‐OMe‐modified oligonucleotide (sequence 8 in Figure 3 c) was not successful, in agreement with previous reports.[^50^, ^51^]

Further, an optional capping step with sodium periodate^[52]^ was possible and could both be used to avoid further ligation of failure sequences (Figure 3 d) or for 3′‐modifications in form of morpholino residues. **RNA1**, bound to streptavidin beads, reacted under mild basic and reductive conditions with a rhodamine derivative containing an amino functional group. The formation of the expected product was confirmed by LC–MS and was also apparent by the purple color of the cleaved and purified RNA (Figure 3 d).

The use of photocleavable linkers can also be of great interest. Within a sequence, they induce strand breaks and thus induce strand dehybridization upon irradiation. This has been used previously in studies where opposite biological effects could be triggered with caged nucleotides and caged strand breaks.[^29^, ^53^, ^54^] We tested whether the 5′‐biotin label can be removed photochemically (Figure 3 e) and whether a photocleavable linker unit can be introduced which provides an internal photocleavage phosphodiester strand break site upon irradiation (Figure 3 f). For the former, we synthesized an RNA fragment with a 5′‐biotin and a photocleavable linker and confirmed that an **RNA1** derivative with this 5′‐modification could be 3′‐extended with **p(*m*Azo)p**. For the latter, we prepared the photocleavable linker bisphosphate **PU** shown in Figure 3 f and confirmed that it could be used for the 3′‐extension of **RNA1**. This extended oligonucleotide could also be ligated to **RNA2** on solid support.

The availability of longer RNAs with multiple modifications will extend RNA chemical biology studies. The current gold standard is chemical solid‐phase synthesis with all its advantages and limitations—such as the fragment length limitation. Herein, we presented a solid‐phase‐based chemoenzymatic alternative based on the 3′‐extension with modified residues and splinted ligation to the next fragment. The tolerance towards modifications is unexpectedly large: We established a whole toolbox for the introduction of fluorophores and especially photocages, photocleavable linkers and photoswitches. Yields for the enzymatic 3′‐extension and the splinted ligation were optimized. The fact that these are significantly lower than the ones of a chemical solid‐phase synthesis using phosphoramidites poses now the next limit—beyond the previous one of a chemical solid‐phase synthesis alone. Our study shows nicely that it is not by the choice of strictly one of the synthesis domains (purely chemical solid‐phase synthesis or purely chemoenzymatic synthesis) but rather by the combination of both domains, where each of them is strong, that the best results are obtained.

## Conflict of interest

The authors declare no conflict of interest.

## Supporting information

As a service to our authors and readers, this journal provides supporting information supplied by the authors. Such materials are peer reviewed and may be re‐organized for online delivery, but are not copy‐edited or typeset. Technical support issues arising from supporting information (other than missing files) should be addressed to the authors.

Supporting Information
