# Supplementary material for: Solid‐Phase‐Supported Chemoenzymatic Synthesis of a Light‐Activatable tRNA Derivative
Source: Angew Chem Int Ed Engl. 2021 Nov 22;61(1):e202111613. doi: 10.1002/anie.202111613 (PMC9299214; doi:10.1002/anie.202111613)
Supplement: Supplementary file 1 — Supporting Information [file ANIE-61-0-s001.pdf]

Angewandte Chemie
Eine Zeitschrift der Gesellschaft Deutscher Chemiker

Supporting Information

Solid-Phase-Supported Chemoenzymatic Synthesis of a Light-Activatable tRNA Derivative

Anja Blümler, Harald Schwalbe, and Alexander Heckel**

anie_202111613_sm_miscellaneous_information.pdf

Table of Contents

Table of Contents 2
Experimental Procedure 3
1. Enzymes used in this study 3
2. Preparation of the magnetic streptavidin beads and buffer exchange 3
3. Enzymatic 3'-extension 4
3.1. Comparison of the 3'-extension in solution and bound to beads 4
3.2. Enzymatic 3'-extension with NPE-modified 3',5'-bisphosphates (pN^{NPE}p) 4
3.3. Enzymatic 3'-extension with azobenzene *C*-nucleoside 3',5'-bisphosphates (p(*m*Azo)p) 6
4. Enzymatic 3'-dephosphorylation 8
5. Enzymatic rephosphorylation using *T4* polynucleotide kinase 9
6. Splinted ligations 9
6.1. Splinted ligations with pre-extended RNA fragments 11
6.2. RP-HPLC purification and mass spectra of the multiple modified NPE-tRNA derivative 13
7. Test ligation with pC^{NPE}p and p(*m*Azo)p within one sequence 14
8. Test ligations with pC^{NPE}p and pU^{DEACM}p 15
9. Synthesis of DEACM-caged uridine bisphosphate 17
9.1. Solvents, reagents and conditions 18
9.2. Synthesis of 2',3',5'-tris-*O*-(*tert*-butyldimethylsilyl)-*O*^{4}-{[7-(diethylamino)-coumarin-4-yl]methyl} uridine (2) 18
9.3. Synthesis of *O*^{4}-{[7-(diethylamino)-coumarin-4-yl]methyl} uridine (3) 18
9.4. Synthesis of 3',5'-*O*-Bisphosphate-*O*^{4}-{[7-(diethylamino)-coumarin-4-yl]methyl} uridine pU^{DEACM}p 19
9.5. NMR spectra 19
9.6. Test ligation of pU^{DEACM}p 21
10. Test ligations with DNA/RNA chimera 21
11. Test ligations with 2'OMe substituted RNA 23
12. Tested sodium periodate capping and 3'-modification with a rhodamine derivative 23
13. Test ligations with a photocleavable biotin residue and p(*m*Azo)p 24
14. Test ligations with an NPE-cleavable 5',3'-diphosphorylated dinucleotide 25
15. Summarized Ligation Yields 27
16. Chemical Solid-Phase Synthesis 27
16.1. Synthesized Sequences 28
16.2. Mass spectra and analytical RP-HPLC chromatograms 29
References 33

Experimental Procedure

1. Enzymes used in this study

Enzymes in this study are all commercially available (see Table S1). *T4 RNA Ligase 1* (Rnl1), *Shrimp Alkaline Phosphatase* (rSAP), and *T4 Polynucleotide Kinase* (PNK) and the appropriate buffers were purchased from *NEB*. The *TURBO^{TM} DNase* used was from *Thermo Fisher*. *T4 RNA Ligase 2* (Rnl2) was homemade, but could also be purchased from *NEB*.

Table S1. Overview of enzymes and conditions used in this publication.

Enzyme	*T4* RNA Ligase 1 (ssRNA Ligase)	Shrimp Alkaline Phosphatase	*T4* Polynucleotide Kinase	*T4* RNA Ligase 2 (dsRNA Ligase)	TURBO^{TM} DNase
**Application**	3'-Ex	3'-DP	5'-P + 3'-DP	Splinted Ligation	DNase Digest
**Vendor**	*NEB*	*NEB*	*NEB*	*Homemade*	*Thermo Fisher*
**Reaction Time**	3 – 4 h	1 - 3 h	2 h	2 – 4 h	30 min
**Reaction Temp**	27 - 37 °C	37 °C	37 °C	17 – 37 °C	37 °C
**Yields**	23 – 93% (depending on fragments)	quantitative	quantitative	20 – 82% (depending on fragments)	quantitative

2. Preparation of the magnetic streptavidin beads and buffer exchange

The magnetic streptavidin beads (4 mg/mL) used were purchased from *New England Biolabs (NEB)*. The beads consist of 1 µm superparamagnetic particles which are covalently coupled with a highly pure form of streptavidin. These beads were delivered in storage buffer containing 0.1% BSA, 0.05% Tween$^{(R)}$ 20, 0.05% NaN_3 in 1x PBS buffer, pH 7.4. The preparation of the magnetic streptavidin beads was performed according to standard protocols also from *NEB*. Before use, the beads were gently vortexed. The required volume of beads (typically 250-800 µL, depending on reaction scale) was transferred to a new sterile microcentrifuge tube. Since the size of the oligonucleotide to be immobilized affects the binding capacity, the amount of streptavidin beads used should exceed the calculated minimum quantity. We usually used a 10% excess of the required beads. The beads were pulled to the wall of the tube with a magnet and the clear supernatant was removed. To wash the beads, they were resuspended in binding buffer (10x the volume of aliquot used, buffer: 20 mM Tris-HCl (pH 7.5), 0.5 M NaCl, 1 mM EDTA). The beads were then pulled again to the wall of the tube and the buffer was removed. This procedure was repeated three times to ensure the complete removal of the storage buffer. After the washing steps, the biotinylated RNA (**RNA1**) dissolved in water was added to the beads and incubated for 20 minutes at 37 °C and 300 rpm. The beads were again pulled to the wall of the tube using the magnet and the supernatant was removed. Again, the beads were washed three times with binding buffer. To confirm that the entire amount of biotinylated RNA had bound to the beads, the OD_{A260} of the supernatant was checked via *NanoDrop* after the incubation and during the washing steps. Finally, the beads with the bound RNA were resuspended in the required volume of water and the appropriate buffers were added for the enzymatic reactions. If fresh/additional beads were added during the reaction pathway, the washing procedure was also repeated.
For buffer exchange, the beads were pulled aside with a magnet for 2 minutes. The clear supernatant (buffer solution and residues from the reaction) was removed and the beads were washed with binding buffer. After that, the beads were resuspended in the required volume of water for the next reaction step before buffer and enzymes were added.
To release modified products from the beads, the reaction buffer was removed. The beads with bound RNA were then resuspended in 100-200µL deionized water and heated to 75 °C for 10 min or 95 °C for 2 min.

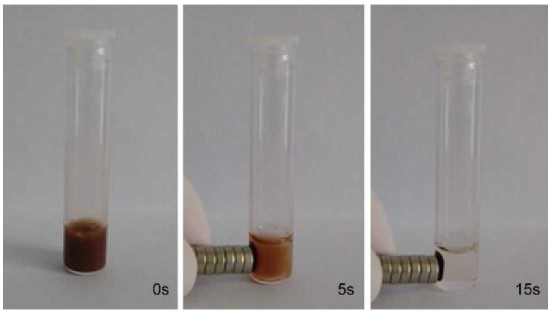

Figure S1. Left: Suspension of magnetic streptavidin beads in aqueous buffer solution. Middle: Beads begin to accumulate on the vial wall due to the attraction of the magnet (picture after 5 s). Right: Beads completely pulled to the side after 15 s.

3. Enzymatic 3'-extension

The incorporation of modifications is based on the enzymatic 3'-extension of an acceptor RNA by a single modified nucleoside 3',5'-bisphosphate using *T4* RNA ligase 1 (Rnl1). Subsequently, either a 3'-dephosphorylation with Shrimp Alkaline Phosphatase (rSAP, within the linear reaction pathway) or a 5'/3'-rephosphorylation with *T4* Polynucleotide Kinase (PNK, in case of pre-extensions) was performed.
The enzymatic 3'-extensions using *T4* RNA Ligase 1 were performed following protocols from *NEB*. We typically used 5-50 µM RNA, 75-150 µM modified bisphosphates (**pC^{NPE}p**, **pU^{NPE}p**, **pG^{NPE}p**, **p(*m*Azo)p**), 1x RNA ligase buffer (50 mM Tris-HCl, 10 mM $MgCl_2$, 1 mM DTT, pH 7.5), 1mM ATP and 5-15% (v/v) *T4* RNA ligase 1 (10000 U/mL). 3'-extensions in solution were performed with additional 10% (v/v) DMSO. Reaction volumes of 20-500 µL were used. Best results were achieved by performing the extensions with a 1:3 ratio of RNA:pNp in small volumes with lower concentrations. Larger scales often lead to a decreased ligation efficiency. For ligation, the reaction mixture was incubated at 37 °C/300 rpm for 3-8 h depending on used pNp and the acceptor RNA. In Figure S2 the structures of the incorporated 3',5'-bisphosphates are shown.

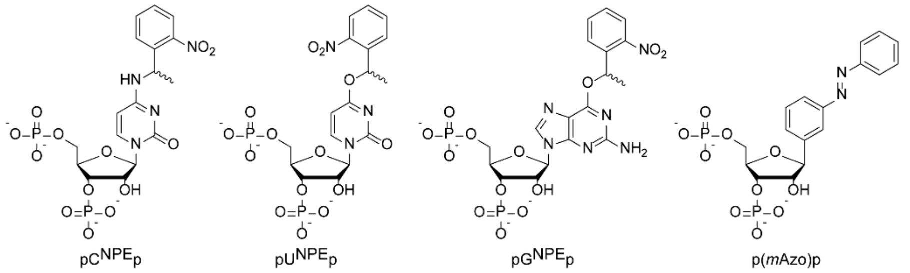

Figure S2. Structure of 3',5'-bisphosphates incorporated enzymatically in this study.

3.1. Comparison of the 3'-extension in solution and bound to beads

In order to assess the influence of the beads on the enzymatic 3'-extension, a reaction in solution (without beads) and a ligation reaction bound to beads were performed under the same conditions. We could not detect any interferences with the reaction process. In Figure S3 20% denaturing polyacrylamide gels of the tested ligations are shown.

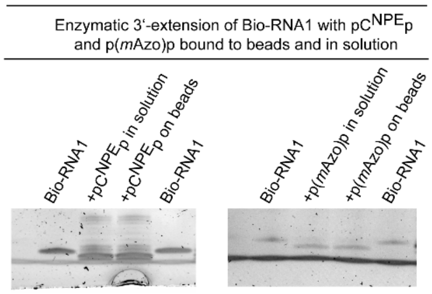

Figure S3. 20% Denaturing polyacrylamide gels showing the tested 3'-extensions of **RNA1** with **pC^{NPE}p** (left) and **p(*m*Azo)p** (right). Both extensions were tested in solution (without beads) and bound to magnetic streptavidin beads. No significant difference could be identified. Products were identified by ESI-MS.

3.2. Enzymatic 3'-extension with NPE-modified 3',5'-bisphosphates (pN^{NPE}p)

For analytical reasons, a reaction aliquot of each 3'-pre-extension was separated via RP-HPLC and the resulting product was analyzed by mass spectrometry. RP-HPLC was performed at 60 °C using an *Agilent 1200 series* equipped with an *XBridge Peptide BEH C18* column (300 Å, 3.5 µm, 4.6x250 mm, detected at 254 nm) from *Waters*. The gradient is shown in Table S2.

Table S2. Gradient used for RP-HPLC analysis.

**Time** [min]	**% solvent A** (MeOH)	**% solvent B** (400 mM HFIP, 16.3 mM Et_3N, pH 8.0)	**Flow rate** [mL/min]
0	5	95	
2	5	95	0.7
30	45.5	54.5	
32	100	0	

SUPPORTING INFORMATION

RP-HPLC chromatograms of the ligation reaction mixtures (right) and measured mass spectra of the products (left) are shown below. Yields were calculated by the integration of corresponding RP-HPLC signals. Starting materials and product peaks are assigned within the chromatograms. LC-ESI mass spectra were recorded on a *Bruker micrOTOF-QII* device. Sequences, as well as calculated and measured masses, are given in Table S3. A 20% denaturing polyacrylamide gel showing the extension of each fragment is also given at the end of this section (see Figure S4).

Table S3. Sample names, corresponding sequences with 3’-terminal NPE-modification, and calculated and measured masses of the isolated ligation products.

Sample	Sequence	Exact calcd Mass [Da]	Measured Mass [Da]
**5’Bio-RNA1-C^{NPE}-p**	5’-**Bio**-r[GGC UAC GUA G**C**^{NPE}]-p-3’	4169.7	4169.7
**5’OH-RNA2-U^{NPE}-p**	5’-r[UCA GUU GGU UAG AGC ACA **U**^{NPE}]-p-3’	6288.8	6289.7
**5’OH-RNA3-C^{NPE}-p**	5’-r[CAC UCA UAA UGA UGG GGU CA**C**^{NPE}]-p-3’	6922.0	6923.0
**5’OH-RNA4-G^{NPE}-p**	5’-r[AGG UUC GAA UCC CGU C**G**^{NPE}]-p-3’	5628.8	5629.6
**5’OH-RNA3^{NPE}-RNA4^{NPE}-p**	5’-r[CAC UCA UAA UGA UGG GGU CA**C**^{NPE} AGG UUC GAA UCC CGU C**G**^{NPE}]-p-3’	12532.7	12536.0

Bio-RNA1 + pC^{NPE}p

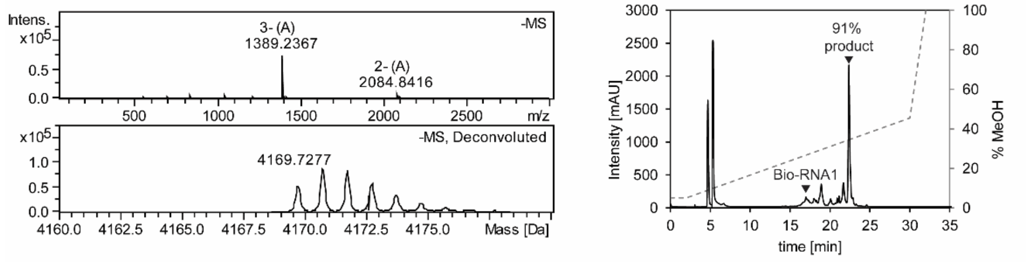

5’OH-RNA2 + pU^{NPE}p

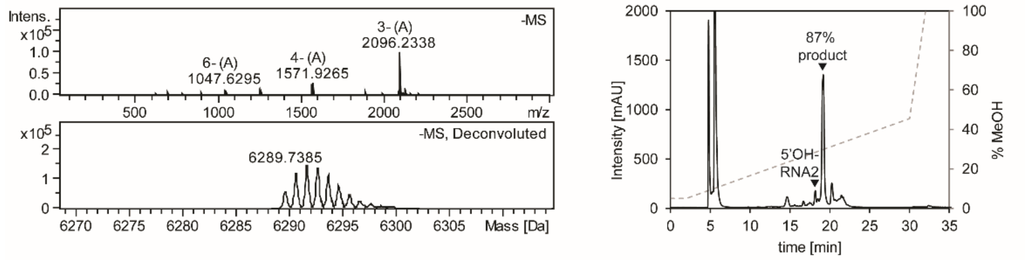

5’OH-RNA3 + pC^{NPE}p

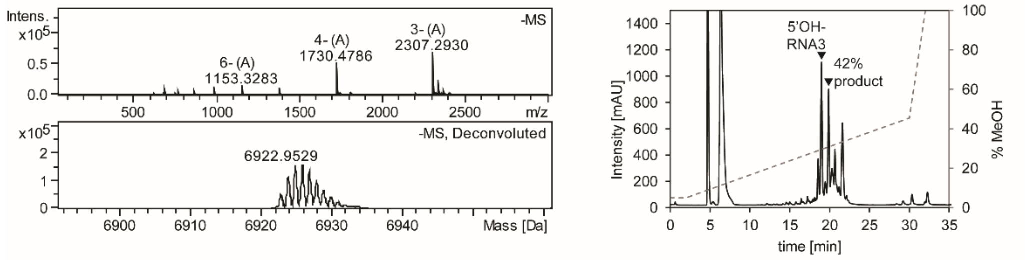

5’OH-RNA4 + pG^{NPE}p

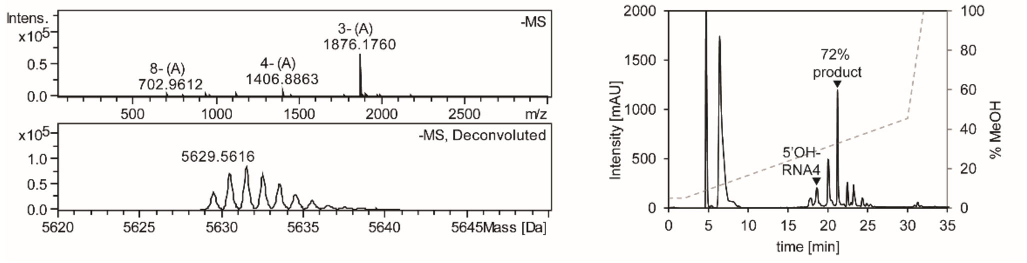

5’OH-RNA3^{NPE}-RNA4 +pG^{NPE}p

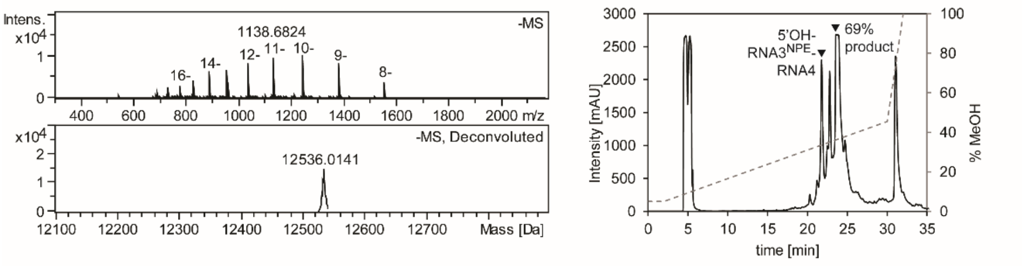

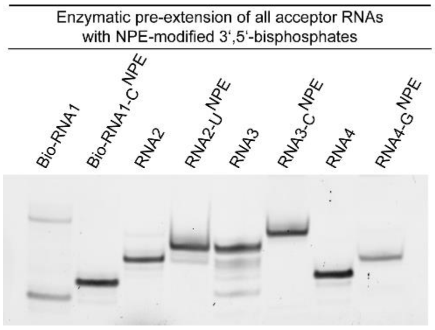

Figure S4. 20% Denaturing polyacrylamide gel showing the 3’-pre-extension with **pN^{NPE}p** of every single RNA fragment. Ligations of the RNA fragments with **pC^{NPE}p**, **pU^{NPE}p,** and **pG^{NPE}p** resulted in 3’-terminal photocaged (**RNA-N^{NPE}-p**) extended RNAs.

3.3. Enzymatic 3’-extension with azobenzene *C*-nucleoside 3’,5’-bisphosphates (p(*m*Azo)p)

For analytical reasons, a reaction aliquot of each 3’-pre-extension was separated via RP-HPLC and the resulting product was analyzed by mass spectrometry. RP-HPLC was again performed using the conditions mentioned before (see Table S2) LC-ESI mass spectra were recorded on a *Bruker micrOTOF-QII* device. Sequences, as well as calculated and measured mass, are given in Table S4. RP-HPLC chromatograms of the ligation reactions and measured mass spectra are shown below. Yields were calculated by the integration of the corresponding RP-HPLC signals. For this, starting materials and product signals are assigned in the chromatograms. A 20% denaturing polyacrylamide gel showing the extension of each fragment is also given at the end of this section (see Figure S5).

Table S4. Sample names, corresponding sequences with 3’-terminal azobenzene C-nucleoside modification, and calculated and measured masses of the isolated ligation products.

Sample	Sequence	Exact calcd Mass [Da]	Measured Mass [Da]
**5’Bio-RNA1-*m*Azo-p**	5’-**Bio**-r[GGC UAC GUA G ***m*Azo**]-p-3’	4092.7	4091.7
**5’OH-RNA2-*m*Azo-p**	5’-r[UCA GUU GGU UAG AGC ACA ***m*Azo**]-p-3’	6209.8	6209.6
**5’OH-RNA3-*m*Azo-p**	5’-r[CAC UCA UAA UGA UGG GGU CA ***m*Azo**]-p-3’	6843.9	6844.8
**5’OH-RNA4-*m*Azo-p**	5’-r[AGG UUC GAA UCC CGU C ***m*Azo**]-p-3’	5510.1	5511.5
**5’OH-RNA3-*m*Azo-RNA4-*m*Azo-p**	5’-r[CAC UCA UAA UGA UGG GGU CA ***m*Azo** AGG UUC GAA UCC CGU C ***m*Azo**]-p-3’	12326.4	12325.2

Bio-RNA1 + p(*m*Azo)p

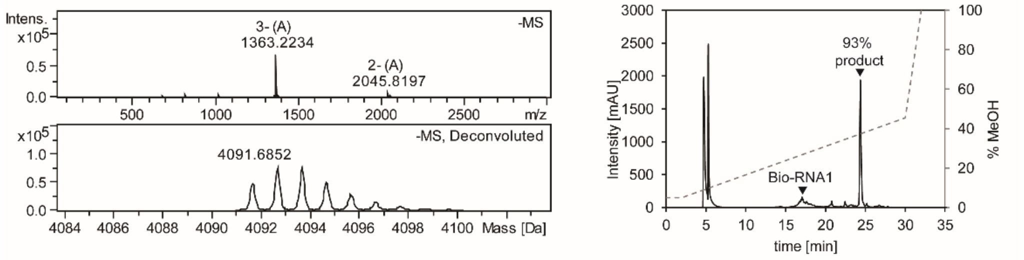

5’OH-RNA2 + p(*m*Azo)p

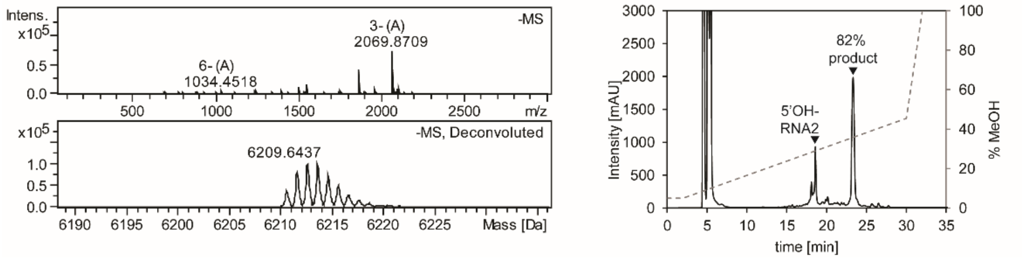

5’OH-RNA3 + p(*m*Azo)p

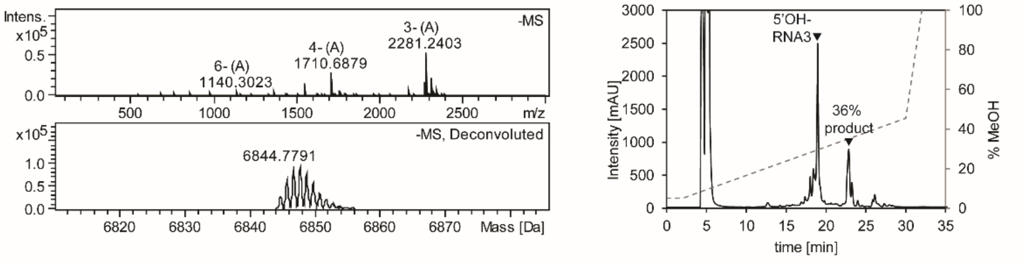

5’OH-RNA4 + p(*m*Azo)p

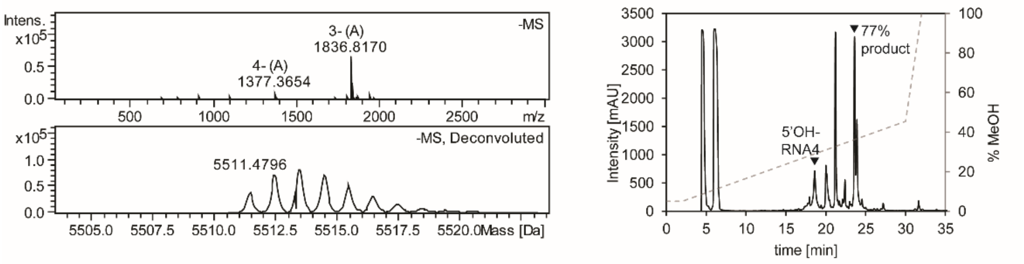

5’OH-RNA3-*m*Azo-RNA4 + p(*m*Azo)p

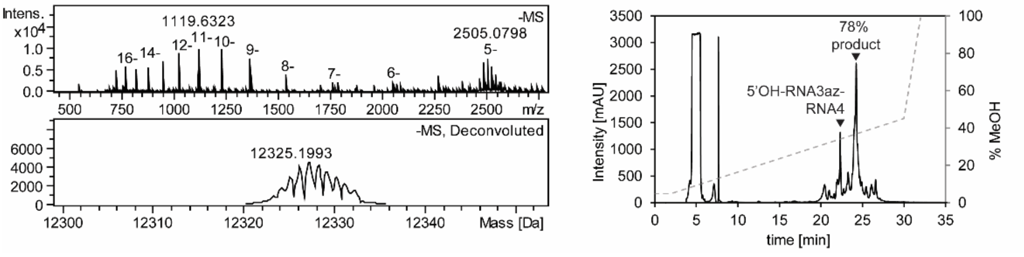

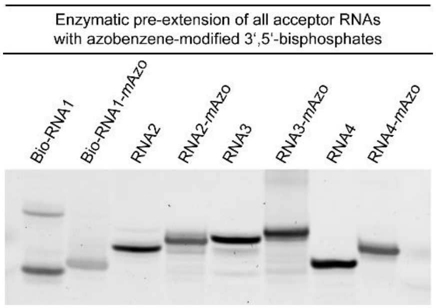

Figure S5. 20% Denaturing polyacrylamide gel showing the 3’-pre-extension with **p(*m*Azo)p** of every single RNA fragment. Ligations of the RNA fragments with **p(*m*Azo)p** resulted in 3’-terminal modified (**RNA-(*m*Azo)-p**) extended RNAs.

4. Enzymatic 3'-dephosphorylation

The enzymatic 3'-dephosphorylation was performed using Shrimp Alkaline Phosphatase (rSAP) and standard protocols from *NEB.* Typical reaction scales ranged from 5 µM up to 50 µM 3'-extended RNA using 1x *Cut Smart Buffer* (50 mM KOAc, 20 mM Tris-acetate, 10 mM $Mg(OAc)_2$ 100 µg/mL BSA, pH 7.9) and 10% (v/v) rSAP (1000 U/mL). The reaction mixture was incubated for 1-3 h at 37 °C and 300 rpm. For analytical reasons, dephosphorylations of the extended Bio-RNA1 fragment were purified and analyzed via RP-HPLC using the same gradient mentioned before (Table S2) and mass spectrometry. Dephosphorylations could be observed as quantitative. Sequences, as well as calculated and measured masses, are given in Table S5. RP-HPLC chromatograms of the area of interest (starting material **Bio-RNA1-C^{NPE}-p** or **Bio-RNA1-*m*Azo-p**; grey line and dephosphorylated product **Bio-RNA1-C^{NPE}-OH** or **Bio-RNA1-*m*Azo-OH**; black line) and measured mass spectra are presented below. A comparison of the dephosphorylation reaction in solution and bound to beads is presented in a 20% denaturing polyacrylamide gel in Figure S6.

Table S5. Sample names, sequences, calculated and measured masses, and RP-HPLC chromatograms of 3'-dephosphorylations.

Sample	Sequence	Exact calcd Mass [Da]	Measured Mass [Da]
**5'Bio-RNA1-C^{NPE}-OH**	5'-**Bio**-r[GGC UAC GUA G **C^{NPE}**]-OH-3'	4090.3	4091.7
**5'Bio-RNA1-*m*Azo-OH**	5'-**Bio**-r[GGC UAC GUA G ***m*Azo**]-OH-3'	4012.7	4011.7

Bio-RNA1-C^{NPE}-p +rSAP

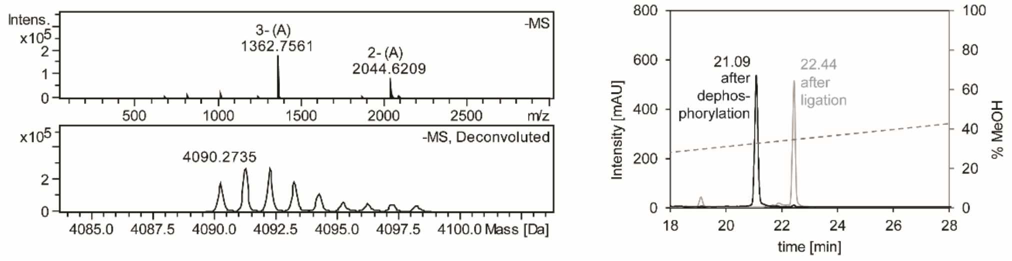

Bio-RNA1-*m*Azo-p +rSAP

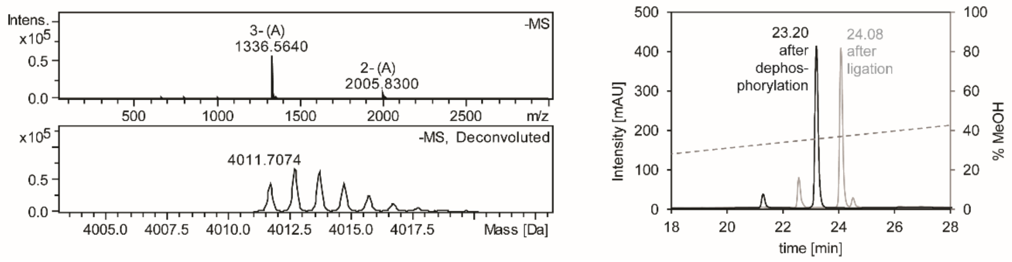

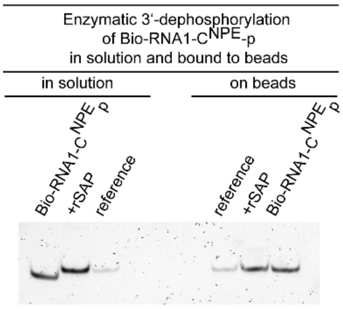

Figure S6. 20% Denaturing polyacrylamide gel showing the 3'-dephosphorylation of Bio-RNA1-C^{NPE}-p with Shrimp Alkaline Phosphatase. The reaction was tested in solution (without beads, left) and bound to magnetic streptavidin beads (right). Dephosphorylated reference samples and measured mass spectra confirm the resulted product.

5. Enzymatic rephosphorylation using *T4* polynucleotide kinase

Rephosphorylations were performed with *T4* polynucleotide kinase (PNK) from *NEB*. Standard protocols and conditions were used (*NEB*). Since PNK catalyzes the phosphorylation of terminal 5'-hydroxyl groups and simultaneously removes the terminal 3' monophosphates, these two reaction steps can be performed at once. To determine the required reaction times, 5'OH-RNA2-U^{NPE}-p was first dephosphorylated with rSAP. After that, the RNA was 5'-phosphorylated with PNK. Typically, reaction scales varied from 5 µM up to 50 µM. the reaction was performed in 1x *T4 PNK Reaction Buffer* containing 70 mM Tris-HCl, 10 mM $MgCl_2$, 5 mM DTT, pH 7.6 and 10% (v/v) PNK (10000 U/mL). The reaction mixture was incubated for 2 h at 37 °C and 300 rpm. A tested 5'-phosphorylation of 5 nmol modified RNA2-U^{NPE}-OH showed, that the reaction was already completed after 30 min. The reaction progress was analyzed by gel electrophoresis (see Figure S7). To ensure complete turnover, all further rephosphorylation reactions were performed at 37 °C/300 rpm for 2 h.

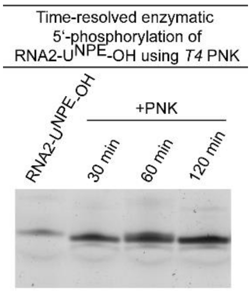

Figure S7. 20% Denaturing polyacrylamide gel of the time-resolved 5'-phosphorylation using *T4* polynucleotide kinase. Samples of the phosphorylation reaction were taken and analyzed after 30, 60, and 120 min. The reaction was already completed after 30 min.

6. Splinted ligations

Splinted ligations were performed on streptavidin beads as solid-phase otherwise it is mentioned in the corresponding section. For solid-phase supported ligations, the first biotinylated RNA fragment (**Bio-RNA1**) was bound to freshly prepared beads as described above (see Section 2). In general, the conditions for all splinted ligations were tested and adjusted for each fragment to be ligated. In particular, the ratios of the fragments and the splint to each other, the reaction temperature and time, and the buffer used for the ligation were varied. Especially the upscaling of ligation approaches proved to be problematic. Therefore, several small reaction mixtures were ligated in parallel (20-150 µl). Typically, RNAs and DNA splints in 2-10 µM concentrations were used. Reaction times varied between 2-4 h. The best results were obtained after pre-annealing of the 5'-phosphorylated donor RNA used to the respective DNA splint before addition to the bound RNA and the streptavidin beads. The fragments and splints used were prepared via chemical solid-phase synthesis and purified (see Section 16). The splinted ligations were performed using *T4* RNA Ligase 2 (homemade, 2 mg/mL) and *T4* DNA ligase buffer. It was found that this buffer resulted in better ligation yields than the recommended *T4* RNA Ligase 2 buffer in most cases. An overview of the RNA fragments and DNA splints used is given in Figure S8.

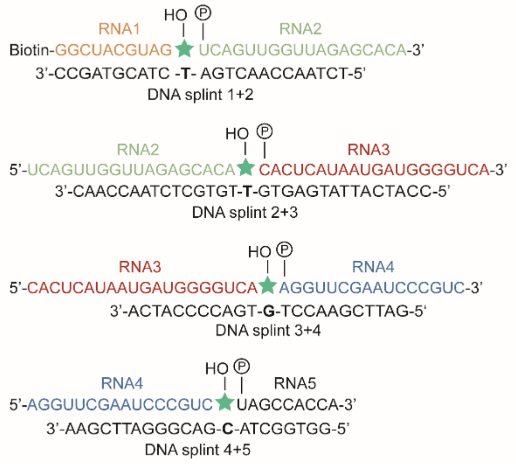

Figure S8. Overview of RNA fragments and corresponding DNA splints used.

Test ligations showed, that there is no significant difference between ligations performed in solution (without beads) or ligations with RNA bound to magnetic streptavidin beads (see Figure S9). For this test, the ligation reactions were performed with a final volume of 100 µL. A 1:1:1 mixture of **Bio-RNA1-C^{NPE}**, **5'p-RNA2,** and a fully complementary **DNA splint** (4 µM each) dissolved in 1x *T4* DNA

ligase buffer (50 mM Tris-HCl, 10 mM $MgCl_2$, 10 mM DTT, pH 7.5 with 1 mM ATP as cofactor) was heated to 80 °C/300 rpm for 4 min. The mixture was then cooled to 37 °C within 10 minutes to pre-anneal the oligonucleotides. In the case of the solid-phase supported ligation, only the 5'-phosphorylated RNA and the DNA splint were pre-annealed before adding them to the suspension of bound RNA and beads. For the splinted ligation, 5% (v/v) homemade *T4* RNA Ligase 2 was added to each and both mixtures were incubated for 3 h at 37 °C/300rpm. A 20% denaturing polyacrylamide gel of these reaction mixtures after 3 h ligation time and before DNase digest can be seen in Figure S9a. Figure S9b,c shows the time course of the ligation reaction in solution and bound to beads.

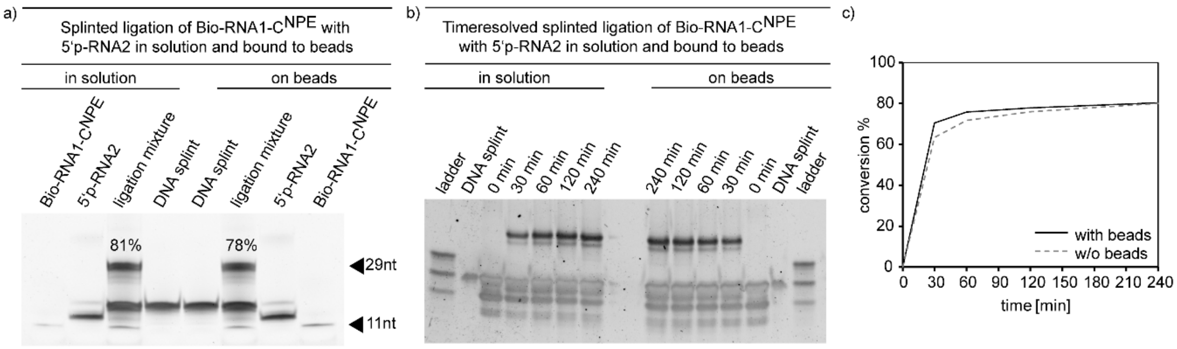

Figure S9. a) 20% Denaturing polyacrylamide gel presenting the comparison of the splinted ligation of NPE-modified RNA1 with the unmodified 5'-phosphorylated RNA2 in solution (left) and bound to beads (right) before DNase digestion. Starting materials and the DNA splint are used to serve as markers. The yields of the internal NPE-modified 29mer are determined by comparison of the gel band intensities using the software *Image Lab*^{TM} (*Bio-Rad*). Yields of 81% in solution and 78% with bound RNA could be observed; b) 20% Denaturing polyacrylamide gel representing the time course of the splinted ligation reaction in solution and bound to beads; c) Plot of the time-resolved ligation conversions.

In general, no significant differences between the final incorporation yields of **p(*m*Azo)p** and **pN^{NPE}p** could be observed. However, the reaction time was different since the 3'-extensions with **p(*m*Azo)p** took approximately 2 hours longer. In Figure S10a the splinted ligation process bound to magnetic streptavidin beads after 1 h reaction time is presented. Reaction mixtures contained a 1:1.5:1 ratio of whether **Bio-RNA1-*m*Azo** or **Bio-RNA1-C^{NPE}**, **5'p-RNA2,** and **DNA splint**. After 1 h only 30% of the starting material **Bio-RNA1-C^{NPE}** can be detected (lane 1). In the case of the azobenzene modified RNA fragment (**Bio-RNA1-*m*Azo**) 68% starting material remain (lane 2). Yields were determined by comparing band intensities of starting materials (Bio-RNA1-Y) with the intensities of product bands. Since the incorporation of **p(*m*Azo)p** is slower than the reaction with the NPE-bisphosphate, different ratios of RNA1:RNA2:splint and the buffer used were tested in splinted ligations of **Bio-RNA1-*m*Azo** and **5'p-RNA2** (see Figure S10b) Best results were achieved with a 1:1:1 ratio and DNA ligase buffer. In Figure S10c, a polyacrylamide gel showing the reaction mixtures of the complete first enzymatic reaction cycle is presented (incorporation of **pC^{NPE}p**). All yields were determined by the comparison of the band intensities using *Image Lab*^{TM}.

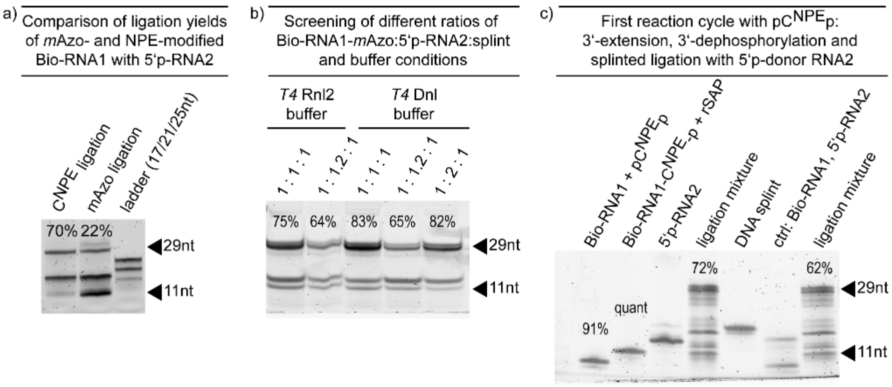

Figure S10. a) Comparison of the ligation yields after a reaction time of 1 h. In lane 1 the splinted ligation of **Bio-RNA1-C^{NPE}** with **5'p-RNA2**; lane 2: 1 the splinted ligation of **Bio-RNA1-*m*Azo** with **5'p-RNA2**; lane 3: RNA ladder (17, 21, 25 nt) as a reference; b) Screening of the first splinted ligation of **Bio-RNA1-*m*Azo** and **5'p-RNA2**; c) 20% denaturing polyacrylamide gel presenting the complete first enzymatic reaction cycle of the incorporation of **pC^{NPE}p**. Lane 1: 3'-extension of **Bio-RNA1** with **pC^{NPE}p** and Rnl1; lane 2: quantitative 3'-dephosphorylation with rSAP; lane 3: 5'-phosphorylated donor **RNA2**; lane 4: ligation mixture after 3 h, lane 5: DNA splint used as a reference, lane 6: a mixture of unmodified starting RNA **Bio-RNA1** and **5'p-RNA2** without ligase as control (ctrl); lane 7: ligation mixture after 2 h reaction time.

In a linear synthesis approach, a subsequent second 3'-extension after the first splinted ligation was performed bound to streptavidin beads. In this step, a second NPE- or *m*Azo-modification was introduced to the 3'-end of **Bio-RNA1-C^{NPE}-RNA2** or **Bio-RNA1-*m*Azo-RNA2** respectively. For this, a buffer exchange was performed after the DNase digestion. The beads were pulled to the wall of the reaction tube and the supernatant was removed. The beads were washed with binding buffer, again pulled to the tube wall to remove the supernatant and resuspended by vortexing in the required volume of water needed for the second 3'-extension with either **pU^{NPE}p** or **p(mAzo)p**. Buffer and bisphosphate were added and the mixture was incubated at 37 °C/300 rpm for 10 minutes before *T4* Rnl1

was added. It turned out, that the second 3'-extension was difficult to quantify and even with this short 29/30mer problematic to confirm the reaction progress properly. Denaturing polyacrylamide gels of both second 3'-extensions (+**pU^{NPE}p** and +**p(*m*Azo)p**) are presented in Figure S11. Ligation yields were determined by the comparison of band intensities.

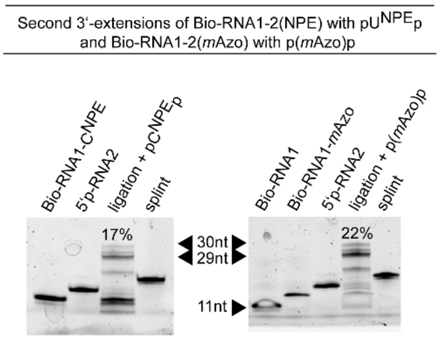

Figure S11. 20% denaturing polyacrylamide gel representing the first splinted ligation followed by a second 3'-extension. Left: Second subsequent 3'-extension of the NPE-modified RNA **Bio-RNA1-C^{NPE}-RNA2** with **pU^{NPE}p**. Lane 1: B**io-RNA1-C^{NPE}** as a reference; lane 2: **5'p-RNA2** as a reference; lane 3: reaction mixture after the splinted ligation of **Bio-RNA1-C^{NPE}** and **5'p-RNA2** and subsequent second 3'-extension with **pU^{NPE}p** resulting in an RNA with two NPE-modifications; lane 4: DNA splint as reference. Right: Second subsequent 3'-extension of the *m*Azo-modified RNA **Bio-RNA1-*m*Azo-RNA2** with **p(mAzo)p**. Lane 1: **Bio-RNA1**; lane 2: B**io-RNA1-mAzo** as a reference; lane 3: **5'p-RNA2** as a reference; lane 4: reaction mixture after the splinted ligation of **Bio-RNA1-mAzo** and **5'p-RNA2** and subsequent second 3'-extension with **p(mAzo)p** resulting in an RNA with two azobenzene modifications; lane 5: DNA splint as reference.

6.1. Splinted ligations with pre-extended RNA fragments

A convergent synthesis strategy was developed to achieve better overall yields. Here, previously in solution 3'-extended and rephosphorylated RNA fragments were solid-phase supported splint ligated (see Sections 3-5). For the subsequent splinted ligations, **Bio-RNA1-C^{NPE}** (same procedure for **Bio-RNA1-*m*Azo** and mAzo-extended RNA fragments respectively) was bound to freshly washed streptavidin beads and incubated at 37 °C/300 rpm for 10 min. **5'p-RNA2-U^{NPE}** was pre-annealed with **DNA splint 1+2** in another reaction tube in *T4* DNA ligase buffer by heating the solution of both oligonucleotides to 80 °C/300 rpm for 4 min. The solution of pre-annealed **RNA2/splint** was then cooled to 37 °C/300 rpm within 10 min and added to the streptavidin bound first RNA fragment (**Bio-RNA1-C^{NPE}**). The first splinted ligations ('**ligation 1**') were performed in final volumes of 100 µL and a 1:1:1 mixture of **Bio-RNA1-C^{NPE}**, **5'p-RNA2-U^{NPE},** and a fully complementary **DNA splint 1+2** (4-8 µM each). 5% (v/v) *T4* RNA ligase 2 was added to the suspension which was then incubated for 3 h at 37 °C/300 rpm. It was also tested whether ligation yields could be further increased by lower reaction temperatures. For this purpose, in addition to reactions at 37 °C/300 rpm aliquots of the previously optimized first splinted ligation were also incubated at 17 °C/300 rpm and 27 °C/300 rpm. The best results were obtained at 37 °C. A denaturing polyacrylamide gel in Figure S12a represents these results. In Figure S12b the first splinted ligation of azobenzene modified RNA fragments can be seen. The measured mass in Figure S12c confirms the splint ligated product of the azobenzene-modified RNA fragments. The measured mass of the NPE-modified product is shown in Figure S16. Yields were determined by a comparison of band intensities.

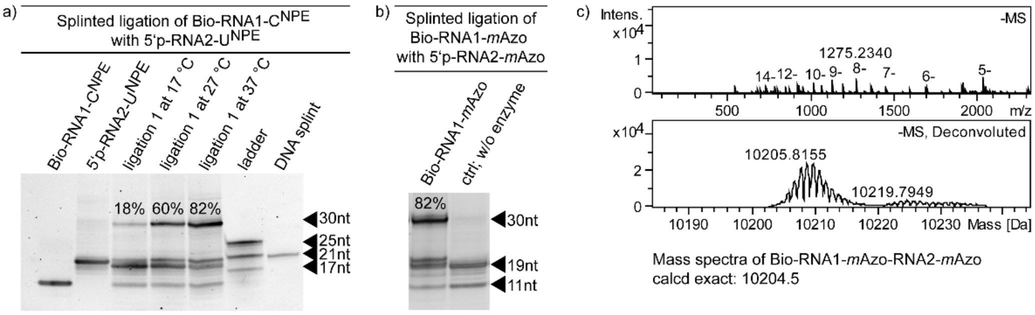

Figure S12. a) 20% Denaturing polyacrylamide gel representing the temperature-dependent ligation yields after 3 h ligation time. Lane 1: pre-extended first RNA fragment **Bio-RNA1-C^{NPE}**; lane 2: pre-extended second donor RNA fragment **5'p-RNA2-U^{NPE}**; lane 3: ligation 1 mixture at 17 °C; lane 4: ligation 1 mixture at 27 °C; lane 5: ligation 1 mixture at 37 °C; lane 6: RNA ladder (17, 21, 25 nt) as a reference; lane 7: **DNA splint 1+2** as a reference. Best yields with 82% were achieved at a ligation temperature of 37 °C. b) Polyacrylamide gel of the first splinted ligation with azobenzene modified RNAs. Lane 1: Mixture of the splinted ligation of **Bio-RNA1-*m*Azo** and **5'p-RNA2-*m*Azo** after 3 h ligation at 37 °C. Lane 2: Control (ctrl) sample: ligation mixture without Rnl1. Yields were determined using *Image Lab^{TM}* c) Measured mass spectra of **Bio-RNA1-*m*Azo-RNA2-*m*Azo**.

After the first splinted ligation, a DNase digestion was performed. Beads were then washed with binding buffer and resuspended in the required proportion of water for the next subsequent splinted ligation. **5'p-RNA3-C^{NPE}** (red RNA fragment in Scheme 1a) was pre-annealed together with **DNA splint 2+3** in the ratio 1.2:1 as described before and added to the suspension of beads and **Bio-RNA1-2 (NPE)** (orange and green RNA fragment in Scheme 1a). The required amounts of **RNA3** and **splint** were calculated based on the starting RNA fragment **Bio-RNA1-C^{NPE}** so that a total calculated ratio of 1:1.2:1 (RNA1-2:RNA3:splint) was used. After the second splinted ligation at 37 °C/300 rpm for 3 h, another DNase digestion was performed (37 °C/300 rpm for 30 min) and the beads were

again washed with binding buffer. The beads with bound **Bio-RNA1-3 (NPE)** were resuspended in the required amount of water for the upcoming third splinted ligation. **5'p-RNA4-G^{NPE}** (blue RNA fragment in Scheme 1a) was pre-annealed together with **DNA splint 3+4** again in the ratio 1.2:1 as described before and added to the suspension of beads and **Bio-RNA1-3 (NPE)** (orange to blue RNA fragment in Scheme 1a). The required amounts of **RNA4** and **splint** were again calculated based on the starting RNA fragment **Bio-RNA1-C^{NPE}** so that a total calculated ratio of 1:1.2:1 (RNA1-3:RNA4:splint) was used. After the third splinted ligation at 37 °C/300 rpm for 3 h, another DNase digestion was performed under the same conditions mentioned before. Figure S13 shows the reaction mixture after the third splinted ligation (**ligation 3**). The yields indicated refer to the respective individual steps (yield for ligation 1, yield for ligation 2 and yield for ligation 3). Yields were determined by comparing the band intensities of the respective ligation products to each other. The respective ligation product from the previous cycle was assumed as starting material for the determination.

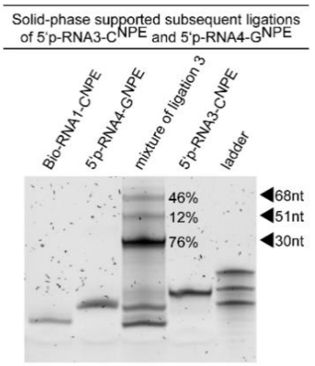

Figure S13. 20% Denaturing polyacrylamide gel after the third subsequent splinted ligation with NPE pre-extended RNA fragments. The third splinted ligation results in an RNA 68mer with 4 NPE modifications. Stepwise yields were determined using *Image Lab^{TM}*. Lane 1: Starting sequence **Bio-RNA1-C^{NPE}** as a reference; lane 2: the last splint ligated 5'-phosphorylated RNA sequence **5'p-RNA4-G^{NPE}** (17 nt); lane 3: ligation mixture of the third subsequent splinted ligation; lane 4: 5'-phosphorylated RNA sequence **5'p-RNA3-C^{NPE}** (21 nt) used in the previous splinted ligation reaction; lane 5: RNA ladder (17, 21, 25 nt) as reference.

Since especially the ligation of **5'p-RNA3-C^{NPE}** resulted in low yields, we decided to prepare a combined RNA fragment **RNA3-C^{NPE}-RNA4** using chemical solid-phase synthesis. This RNA 37mer was also enzymatically 3'-modified, resphophorylated with PNK, and subsequently splint ligated with **Bio-RNA1-2 (NPE)** bound to streptavidin beads. After the first splinted ligation, a DNase digestion was performed. Beads were washed with binding buffer and resuspended in the required proportion of water for the next subsequent splinted ligation as described before. **5'p-RNA3-C^{NPE}-RNA4-G^{NPE}** (combined red and blue RNA fragment in Scheme 1a) was pre-annealed together with **DNA splint 2+3** in the ratio 1.2:1 as described before and added to the suspension of beads and **Bio-RNA1-2 (NPE)** (orange and green RNA fragment in Scheme 1a). The required amounts of **5'p-RNA3-4 (NPE)** and **splint** were calculated based on the starting RNA fragment **Bio-RNA1-C^{NPE}** so that a total calculated ratio of 1:1.2:1 (RNA1-2:RNA3-4:splint) was used. The splinted ligation was performed at 37 °C/300 rpm for 3 h before the DNA splint was digested using *TURBO DNase^{TM}*. The beads with bound **Bio-RNA1-4 (NPE)** were washed with binding buffer and resuspended in water for a third next splinted ligation with the final **5'p-RNA5** (black RNA in Scheme 1a) fragment. **5'p-RNA5** was pre-annealed together with **DNA splint 4+5** again in the ratio 1.2:1 by heating the mixture in *T4* DNA ligase buffer to 80 °C/300 rpm for 4 min. The solution was then cooled to 27 °C/300 rpm within 10 min and added to the streptavidin bound RNA fragment **Bio-RNA1-4 (NPE)**. The last splinted ligation was performed by incubation with 5% (v/v) *T4* Rnl2 at 27 °C/300 rpm for 4 h. The reduced reaction temperature of 27 °C was chosen to allow good hybridization, especially of the short RNA fragment (RNA5, 9nt) with the DNA splint, to support the ligation reaction. After 4 h the DNA splint was digested. The beads were washed, resuspended in 100 µL deionized water and heated to 75 °C for 10 min to release the modified RNA-77mer. The full-length product was analyzed by gel electrophoresis, RP-HPLC and mass spectrometry. Figure S14 represents the results of the gel electrophoresis. Stepwise yields were determined by comparison of band intensities using *Image Lab^{TM}*. The procedure of the final purification and measured masses of the RNA with four internal NPE modifications via RP-HPLC and ESI-LC-MS is described in Section 6.2.

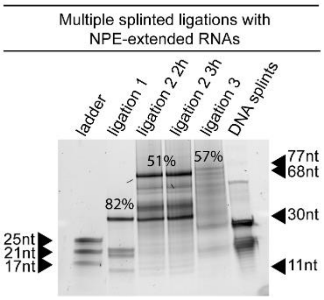

Figure S14. 20% Denaturing polyacrylamide gel representing the solid-phase supported ligation reactions. Lane 1: RNA ladder (17, 21, 25 nt); lane 2: a mixture of ligation 1 (**Bio-RNA1-C^{NPE}** + **5'p-RNA2-U^{NPE}**); lane 3: a mixture of ligation 2 after 2 h reaction time (**Bio-RNA1-C^{NPE}-RNA2-U^{NPE}** + **5'p-RNA3-C^{NPE}-RNA4-G^{NPE}**); lane 4: a mixture of ligation 2 after 3 h reaction time (**Bio-RNA1-C^{NPE}-RNA2-U^{NPE}** + **5'p-RNA3-C^{NPE}-RNA4-G^{NPE}**); lane 5: a mixture of ligation 3 (**Bio-RNA1-C^{NPE}-RNA2-U^{NPE}-RNA3-C^{NPE}-RNA4-G^{NPE}** + **5'p-RNA5**); lane 6: a mixture of the DNA splints used in the respective splinted ligations.

6.2. RP-HPLC purification and mass spectra of the multiple modified NPE-tRNA derivative

The final purification of the modified RNA-77mer was performed by RP-HPLC. Due to the advantages of the solid-phase-based method, only a separation of biotinylated fragments differing in length by several bases is necessary. Purification of sequences with a single base difference (n-1) is thus avoided. By adjusting the gradients used, a separation of the possible fragments could be achieved. RP-HPLC was carried out at 60 °C using an *Agilent 1200 series* equipped with an *XBridge Peptide BEH C18* column (300 Å, 3.5 µm, 4.6x250 mm, 0.7 mL/min, detected at 254 nm) from *Waters*. Solvents were MeOH (solvent A) and HFIP Buffer (400 mM HFIP, 16.3 mM Et_3N, pH 8.0, solvent B). Best separation results were achieved with gradient C. RP-HPLC chromatograms with assigned NPE-modified products and gradients used are shown in Figure S15. The fragments were analyzed by mass spectrometry. Corresponding mass spectra can be seen in Figure S16.

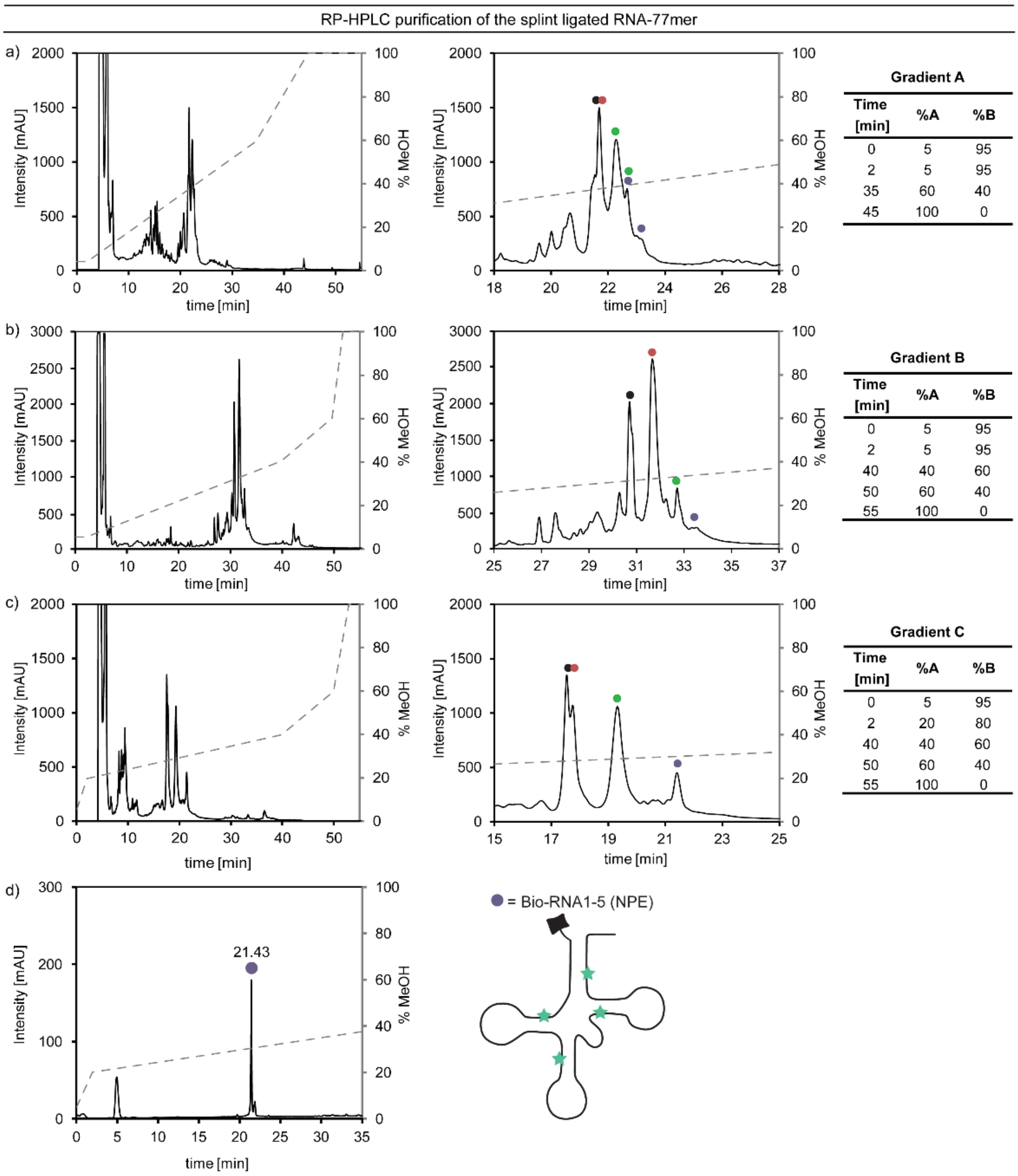

Figure S15. a-c) Optimization of the final RP-HPLC purification (● = RNA1, ● = product of “ligation 1”, ● = product of “ligation 2”, ● = product “ligation 3”). By adjusting the gradient, a good separation of biotinylated fragments could be achieved. Best results can be seen in c). d) Analytical RP-HPLC chromatogram of the purified full-length product. Uridine was used as an internal standard (elution after 5 min).

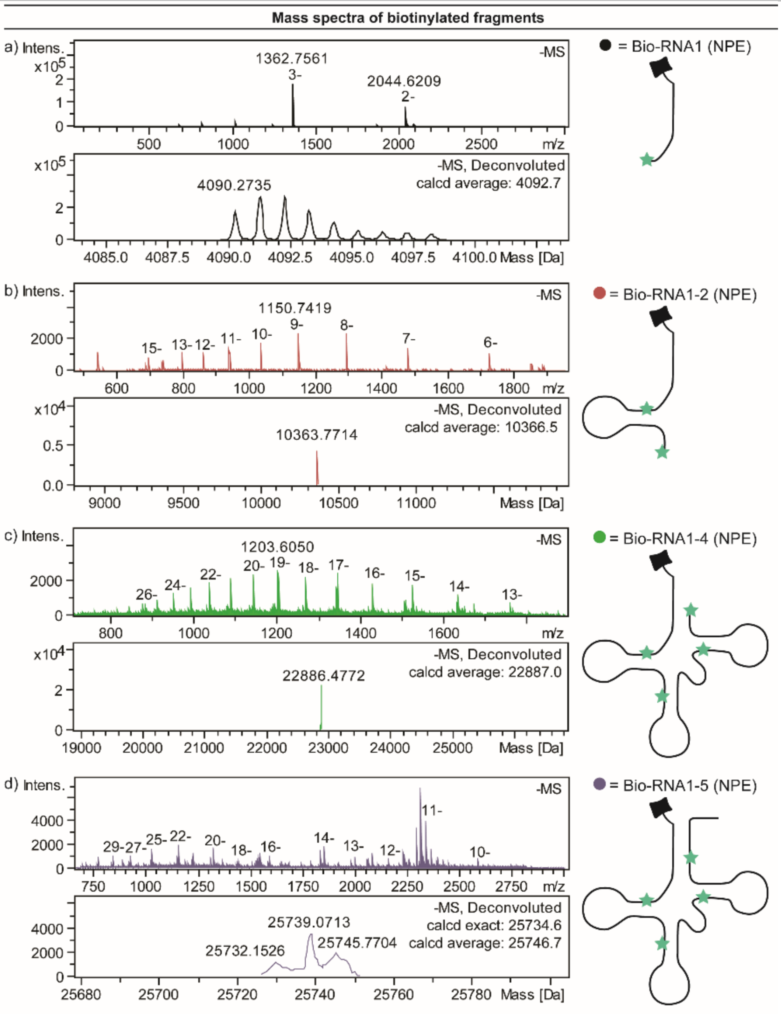

Figure S16. Measured mass spectra of all biotinylated fragments recorded on a *Bruker micrOTOF-QII* device. Measured masses are shown in the deconvoluted mass spectra. Calculated masses are given within the corresponding spectra. The color code corresponds with the one used in Figure S15. a) Mass spectrum of NPE-modified Bio-RNA1 (NPE); b) Mass spectrum of the isolated RNA fragment with two NPE modifications (Bio-RNA1-2 (NPE)); c) Mass spectrum of the isolated RNA with four NPE modifications (Bio-RNA1-4(NPE)); d) Mass spectrum of the fourfold NPE-modified RNA-77mer (Bio-RNA1-5 (NPE)).

7. Test ligation with pC^{NPE}p and p(*m*Azo)p within one sequence

To show, that the synthesis of a mixed modified RNA is possible, we incorporated both modifications (NPE and azobenzene) into one sequence. It should be considered that the *trans/cis* photoisomerization of azobenzene at 365 nm leads to cleavage of the NPE protecting group. Nevertheless, the incorporation of both modifications into one sequence is possible.

A splinted ligation with a 1:2:1 mixture of azobenzene extended **Bio-RNA1-*m*Azo-OH** (8 µM) and **5'p-RNA2-U^{NPE}** (16 µM) in presence of a DNA splint (8 µM) was performed using *T4* Rnl2. The reaction volume was 100 µL in 1x *T4* DNA ligase buffer. The mixture was incubated at 37 °C/300 rpm for 4 h. The ligation mixture was analyzed via denaturing polyacrylamide gel (see Figure S17, yields were determined by analyzing the band intensities using *ImageLab^{TM}*) and purified by RP-HPLC. The purified product could be confirmed via mass spectrometry (see below).

Table S6. Sample name, sequence and calculated and measured mass of the 3'-DEACM-modified RNA2.

Sample	Sequence	Exact calcd Mass [Da]	Measured Mass [Da]
**Bio-RNA1-*m*Azo-RNA2-U^{NPE}**	5'-**Bio**-r[GGC UAC GUA G***m*Azo**U CAG UUG GUU AGA GCA CA**U^{NPE}**]-OH-3'	10283.5	10284.9

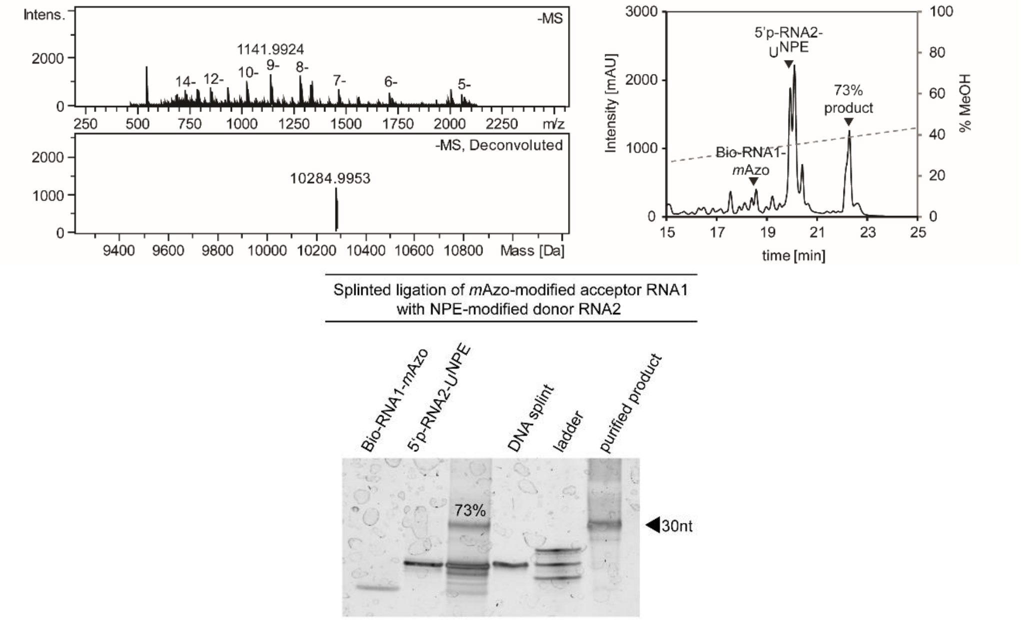

Figure S17. Upper part: Mass spectrum (left) and RP-HPLC chromatogram of the purification (right) are shown. Lower part: 20% denaturing polyacrylamide gel representing the splinted ligation of Bio-RNA1-*m*Azo with 5’p-RNA2-U^{NPE}. Lane1: azobenzene-modified **Bio-RNA1-mAzo**; lane 2: NPE-caged **5’p-RNA2-U^{NPE}**; lane 3: ligation mixture after 4 h; lane 4: DNA splint; lane 5: RNA ladder (17, 21, 25 nt) as a reference; lane 6: RP-HPLC purified ligation product.

8. Test ligations with pC^{NPE}p and pU^{DEACM}p

In order to address multiple caged RNAs wavelength-selectively, we tested the incorporation of a DEACM-modified uridine 3’,5’-bisphosphate. This bisphosphate was synthesized analog to our previously published synthesis route (see Section 9).[1] Due to the harsh basic conditions during the cleavage procedure, after a chemical solid-phase synthesis it is not possible to introduce a DEACM-caged uridine chemically. At this point, chemo-enzymatic incorporation under nearly physiological conditions offers advantages. We tested the 3’-extension of RNA2 with **pU^{DEACM}p**. The reaction was performed in solution in a total volume of 100 µL using 50 µM **RNA2** and 150 µM **pU^{DEACM}p**. A mixture in 1x *T4* RNA ligase buffer with 10% (v/v) DMSO and 5% (v/v) *T4* Rnl1 was incubated at 37 °C/300 rpm for 3 h. To deactivate the enzyme, the mixture was heated to 65 °C for 10 minutes. The reaction was purified via RP-HPLC (gradient Table S2) and the product was confirmed by mass spectrometry. The sequence and calculated and measured mass are given in Table S7. Mass spectra recorded on a *Bruker micrOTOF-QII* device (left) and the RP-HPLC chromatogram of the purification (right) are shown below.

Table S7. Sample name, sequence and calculated and measured mass of the 3’-DEACM-modified RNA2.

Sample	Sequence	Exact calcd Mass [Da]	Measured Mass [Da]
**5’OH-RNA2-U^{DEACM}-p**	5’-r[UCA GUU GGU UAG AGC ACA **U^{DEACM}**]-p-3’	6368.9	6369.8

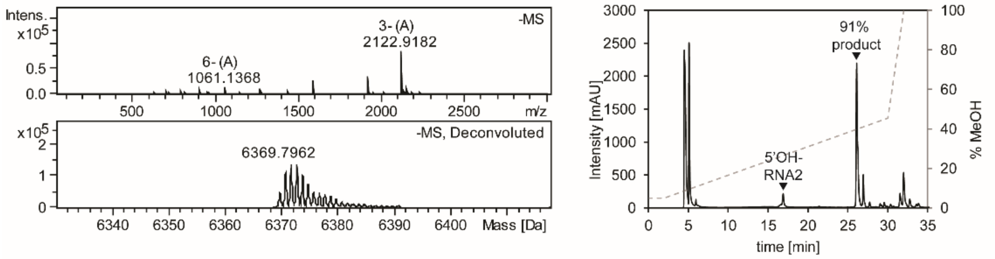

After purification of the DEACM-caged RNA, the solvent was removed at 4 °C using a vacuum concentrator from *Eppendorf*. The purified product **RNA2-U^{DEACM}-p** was rephosphorylated with *T4* PNK. To combine the NPE- and DEACM-modification within one sequence, a splinted ligation with a 1:1:1 mixture of 3’-NPE-extended **Bio-RNA1-C^{NPE}** and the 3’-DEACM-extended **5’p-RNA2-U^{DEACM}** in presence of a DNA splint (each 8 µM) was executed. The total ligation volume was 100 µL in *T4* DNA ligase buffer. The mixture was incubated at 37 °C/300 rpm for 3.5 h. First irradiation experiments were carried out with LEDs from *Thorlabs*. For the selective cleavage of the DEACM-protecting group, an aliquot of the ligation mixture was irradiated at 455 nm for 10 min. The subsequent cleavage of the

NPE-protecting group occurred after a second irradiation at 365 nm for another 10 min. A band shift after each irradiation can be observed. The modified sequence, the structure of **pU^{DEACM}p** and a polyacrylamide gel of the ligation reaction as well as the first irradiation experiments with the ligation mixture can be seen in Figure S18.

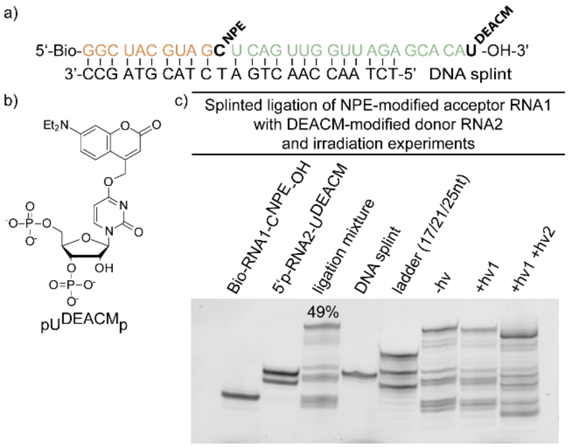

Figure S18. a) Sequence of the NPE- and DEACM-modified RNA fragments and the DNA splint used; b) The structure of the DEACM-modified uridine 3',5'-bisphosphate; c) 20% denaturing polyacrylamide gel representing the splinted ligation as well as the first irradiation experiments. Lane1: NPE-caged **Bio-RNA1-C^{NPE}**; lane 2: DEACM-caged **RNA2-U^{DEACM}**; lane 3: ligation mixture after 3.5 h; lane 4: DNA splint; lane 5: RNA ladder (17, 21, 25 nt) as a reference; lane 6: ligation mixture before irradiation; lane 7: ligation mixture after the first irradiation at 455 nm; lane 8: ligation mixture after the first irradiation at 455 nm and the second irradiation at 365 nm.

To confirm the uncaging process, after DNase digestion, the ligation product was isolated via RP-HPLC and its identity confirmed by mass spectrometry. First, the purified product (100 µL; 5 µM) was irradiated at 455 nm for 3 min and analyzed by mass spectrometry. Then, the NPE-protecting group was cleaved via a second irradiation at 365 nm for 10 min. The irradiation product was again analyzed by mass spectrometry. A peak shift in the RP-HPLC chromatogram after each irradiation can be detected. Calculated and measured masses, as well as the corresponding RP-HPLC chromatograms of the purified product before and after irradiation can be seen in Table **S8** and Figure S19.

Table S8. Sample names, sequences as well as calculated and measured masses of the ligation product before and after irradiation.

Sample	Sequence	Exact calcd Mass [Da]	Measured Mass [Da]
**Bio-RNA1-C^{NPE}-RNA2-U^{DEACM}**	5'-**Bio**-r[GGC UAC GUA G**C^{NPE}**U CAG UUG GUU AGA GCA CA**U^{DEACM}**]-OH-3'	10441.6	10443.0
**Bio-RNA1-C^{NPE}-RNA2-U**	5'-**Bio**-r[GGC UAC GUA G**C^{NPE}**U CAG UUG GUU AGA GCA CA**U**]-OH-3'	10212.5	10212.7
**Bio-RNA1-C-RNA2-U**	5'-**Bio**-r[GGC UAC GUA G**C**U CAG UUG GUU AGA GCA CA**U**]-OH-3'	10063.4	10063.8

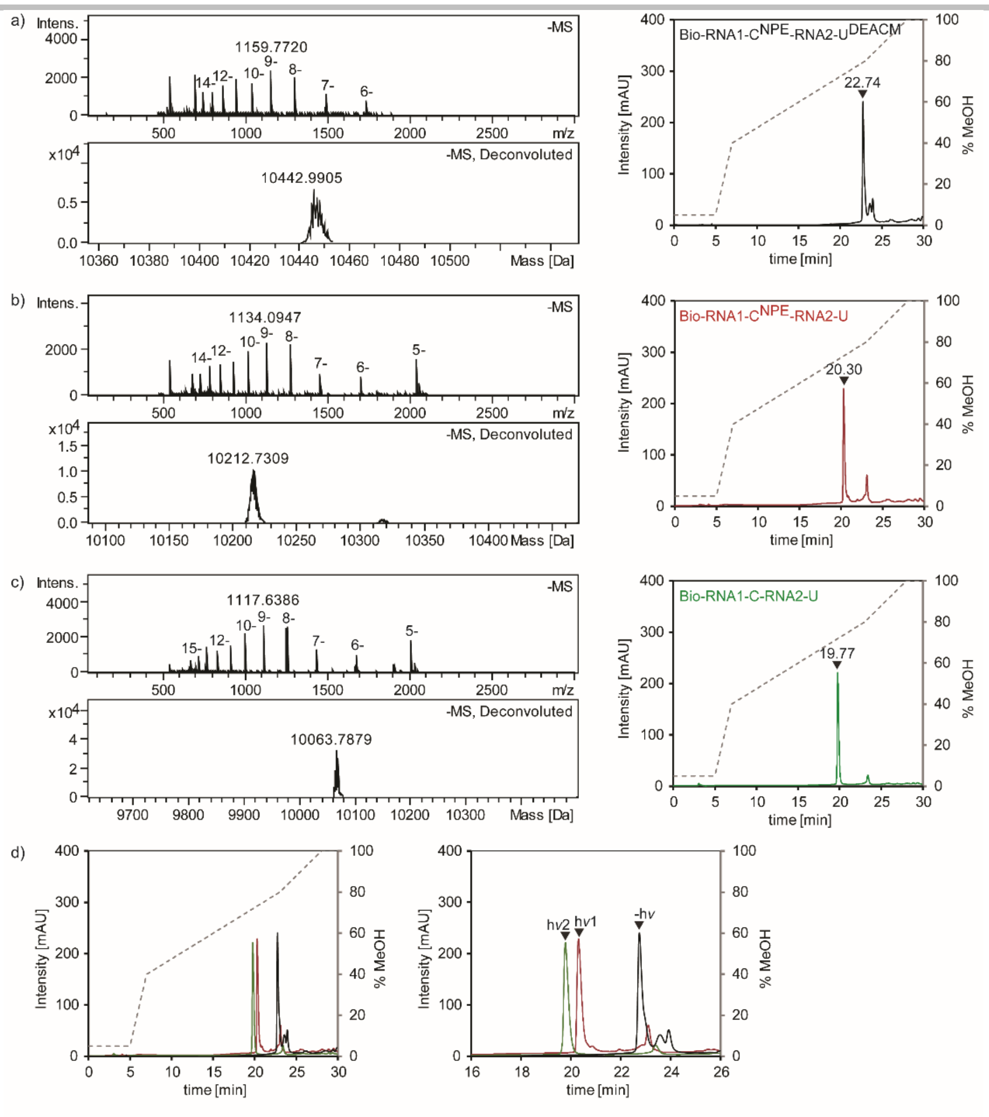

Figure S19. a) Mass spectrum and RP-HPLC chromatogram of the purified ligation product **Bio-RNA1-C^{NPE}-RNA2-U^{DEACM}** before irradiation; b) Mass spectrum and RP-HPLC chromatogram of the ligation product after the first irradiation at 455 nm resulting in **Bio-RNA1-C^{NPE}-RNA2-U**; c) Mass spectrum and RP-HPLC chromatogram of the ligation product after the second subsequent irradiation at 365 nm resulting in **Bio-RNA1-C-RNA2-U**; d) Overlay of the RP-HPLC chromatograms and zoom to the region of interest.

9. Synthesis of DEACM-caged uridine bisphosphate

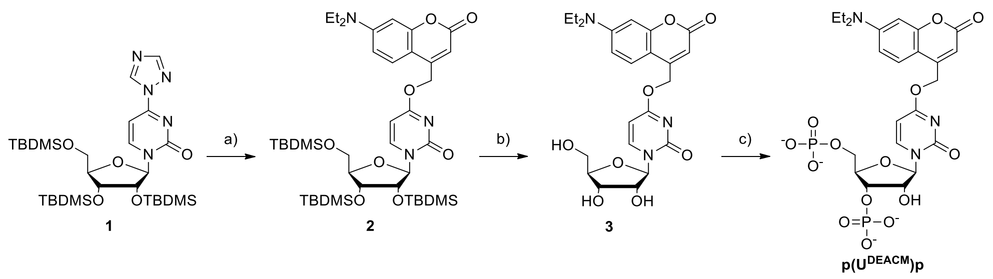

Scheme S1. Overview of the synthesis of the DEACM-caged uridine bisphosphate **pU^{DEACM}p**. Compound **1** was synthesized as described earlier.[1] a) DEACM-OH (prepared according to the literature procedure[2]), DBU, MeCN, rt, 16 h, 52% b) TBAF, AcOH, THF, rt, 8 h, 87%; c) diphosphoryl chloride, -12 °C, 6 h, 1 M TEAB, crude mixture of 2',5'- and 3',5'-bisphosphate.

9.1. Solvents, reagents and conditions

All reactions were performed under argon atmosphere and if necessary under light exclusion. Reagents and dry solvents were purchased from *Acros Organics*, *Sigma Aldrich*, *TCI Chemicals, Alfa Aesar* or *Carbosynth* and used without further purification. The silica gel used for column chromatography was purchased from *Macherey-Nagel* (particle size: 40-63 µm). NMR spectra were recorded on *Bruker DPX250* or *AV400* and *AV500* instruments at ambient temperature. The spectra were assigned to the corresponding solvent peak of DMSO-d_6 at 2.50ppm.

9.2. Synthesis of 2',3',5'-tris-*O*-(*tert*-butyldimethylsilyl)-*O*^{4}-{[7-(diethylamino)-coumarin-4-yl]methyl} uridine (2)

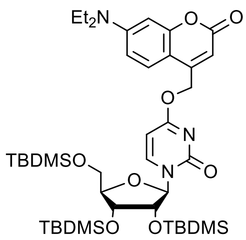

2

Under argon atmosphere and light exclusion 3.00 g of the triazole uridine derivative **2** (4.7 mmol, 1.0 eq) were dissolved in 100 mL dry MeCN to give an orange colored solution. 1.26 mL 1,8-diazabicyclo[5.4.0]undec-7-ene (DBU, 8.5 mmol, 1.8 eq) and 1.28 g 7-(diethylamino)-4-(hydroxymethyl)chromen-2-one (DEACM-OH, 5.2 mmol, 1.1 eq) were added to the solution. The dark orange mixture was stirred at room temperature for 16 h. After evaporation, the crude product was purified by column chromatography (SiO_2, cyclohexane/EtOAc 4:1 → 2:1) to give **2** as a yellow solid.

Yield: 1.98 g (4.7 mmol, 52%)
TLC (cyclohexane/EtOAc 4:1): R_f = 0.40
^{1}H-NMR (DMSO-d_6, 400 MHz) δ = 8.24 (d, ^3J(H,H) = 7.5 Hz, 1H, H5), 7.48 (d, ^3J(H,H) = 9.0 Hz, 1H, DEACM-H5), 6.71 (d, ^3J(H,H) = 9.0 Hz, 1H, DEACM-H6), 6.56 (s, 1H, DEACM-H8), 6.24 (d, ^3J(H,H) = 7.0 Hz, 1H, H6), 6.02 (s, 1H, DEACM-H3), 5.80 (m, 1H, H1'), 5.56 (bs, 2H, O-C$\underline{H_2}$-DEACM), 4.22 (m, 1H, H2'), 4.08 (m, 1H, H3'), 4.00-3.95 (m, 2H, H4' and H5'), 3.76-3.73 (m, 1H, H5'), 3.46-3.41 (m, 4H, 2x C$\underline{H_2}$-CH_3), 1.12 (t, ^3J(H,H) = 6.1 Hz, 6H, 2x CH_2-C$\underline{H_3}$), 0.92-0.83 (m, 27H, TBDMS), 0.12-0.02 (m, 18H, TBDMS)ppm.

9.3. Synthesis of *O*^{4}-{[7-(diethylamino)-coumarin-4-yl]methyl} uridine (3)

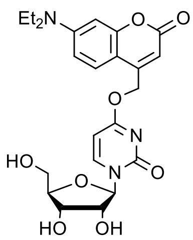

3

1.90 g of DEACM- and TBDMS-protected **2** (2.3 mmol, 1.0 eq) were dissolved in 40 mL dry THF. 8.2 mL TBAF (1.0 M in THF, 8.2 mmol, 3.5 eq) were added and the mixture was stirred at room temperature for 8 h. After evaporation, the crude product was purified via column chromatography (SiO_2, CH_2Cl_2/MeOH 9:1) to give **3** as pale yellow solid.

Yield: 953 mg (2.3 mmol, 86%)
TLC (CH_2Cl_2/MeOH 9:1): R_f = 0.61
^{1}H-NMR (DMSO-d_6, 250 MHz) δ = 8.42 (d, ^3J(H,H) = 7.5 Hz, 1H, H5), 7.48 (d, ^3J(H,H) = 9.0 Hz, 1H, DEACM-H5), 6.74-6.70 (m, 1H, DEACM-H6), 6.57-6.56 (m, 1H, DEACM-H8), 6.24 (d, ^3J(H,H) = 7.0 Hz, 1H, H6), 6.02 (s, 1H, DEACM-H3), 5.80-5.79 (m, 1H, H1'), 5.54 (bs, 2H, O-C$\underline{H_2}$-DEACM), 5.47-5.45 (m, 1H, 2'OH), 5.16 (t, ^3J(H,H) = 5.0 Hz, 3'OH), 5.03-5.00 (m, 1H, 5'OH), 4.00-3.89 (m, 3H, 2'H, 3'H and 4'H), 3.77-3.55 (m, 2H, 5'-C$\underline{H_2}$), 3.45-3.43 (m, 4H, 2x C$\underline{H_2}$-CH_3), 1.13 (t, ^3J(H,H) = 6.9 Hz, 6H, 2x CH_2-C$\underline{H_3}$)ppm.
^{13}C-NMR (DMSO-d_6, 126 MHz) δ = 170.0, 160.7, 155.8, 154.6, 150.5, 145.2, 125.5, 108.8, 105.3, 97.1, 96.9, 94.4, 90.0, 87.7, 84.8, 84.2, 74.5, 73.5, 68.8, 63.2, 59.9, 44.0, 12.3ppm.

9.4. Synthesis of 3',5'-*O*-Bisphosphate-*O*4-{[7-(diethylamino)-coumarin-4-yl]methyl} uridine pU^{DEACM}p

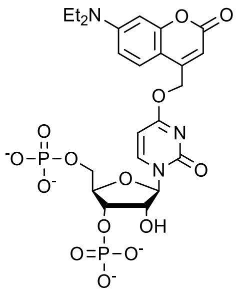

pU^{DEACM}p

The synthesis was performed according to literature procedure.[1,3] 15 mg of **3** (32 µmol, 1 eq) were cooled to -12 °C under argon atmosphere. 44 µL diphosphoryl chloride (317 µmol, 10 eq) were slowely added. The mixture was stirred at -12 °C for 6 h. The reaction was stopped by adding ice to the mixture and adjusting the pH to 7.5 using pre-chilled 1 M triethylammonium bicarbonate buffer (TEAB, pH 8.0). The solvent was removed under reduced pressure. The crude reaction mixture was analyzed by NMR and mass spectrometry. The product could be identified. A further purification via column chromatography or RP-HPLC turned out to be problematic, since the DEACM-protecting group was partially cleaved during these attemps. The crude product (a mixture of 3',5'- and 2',5'-pU^{DEACM}p) was coevaporated multiple times with RNase free water and test ligated using *T4* RNA ligase 1. The enzyme only accepts the 3',5'-phosphorylated compound as substrate.[3] The 3'-extension as well as the 3'-dephosphorylation was successful. Both could be confirmed by mass spectrometry. The crude product was therefore not further purified.

Yield: The crude product could be identified as a mixture of 3',5'-bisphosphate and 2',5'-bisphosphate
RP-TLC (*i*PrOH/NH_3/H_2O 6:3:1): R_f = 0.27
^{31}P-NMR (D_2O, 202 MHz) δ = 1.01, 0.84, 0.64, 0.55ppm.
HRMS (MALDI): m/z calcd for $C_{23}H_{29}N_3O_{14}P_2 + H^+$ [M+H$^+$] 634.11975; found: 634.11876 (Δm= 0.00099; error 1.6ppm).

9.5. NMR spectra

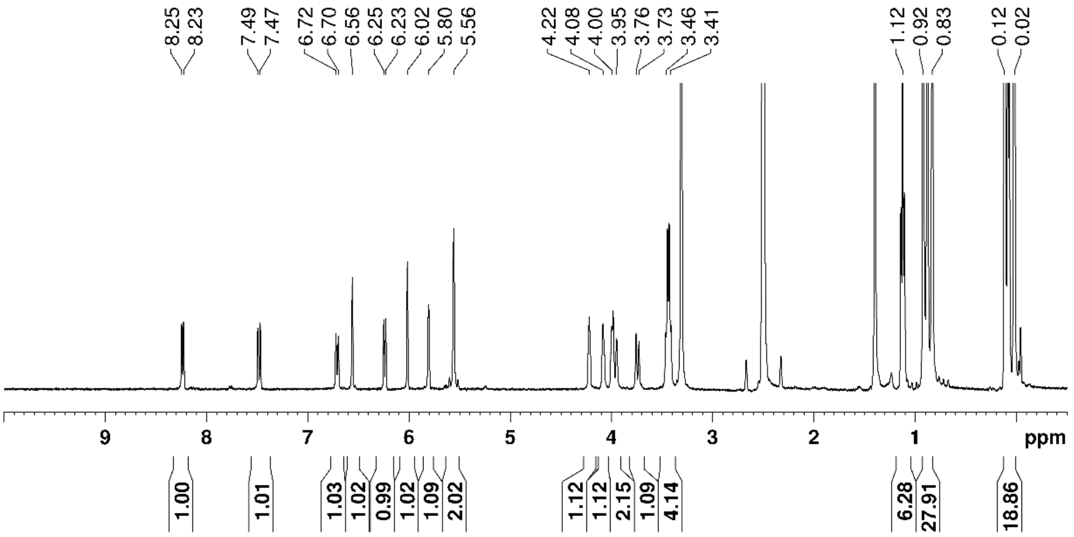

Figure S20. ^{1}H-NMR spectra of compound **2** recorded on a *Bruker AV400* spectrometer in DMSO-d_6 (400 MHz, 298 K).

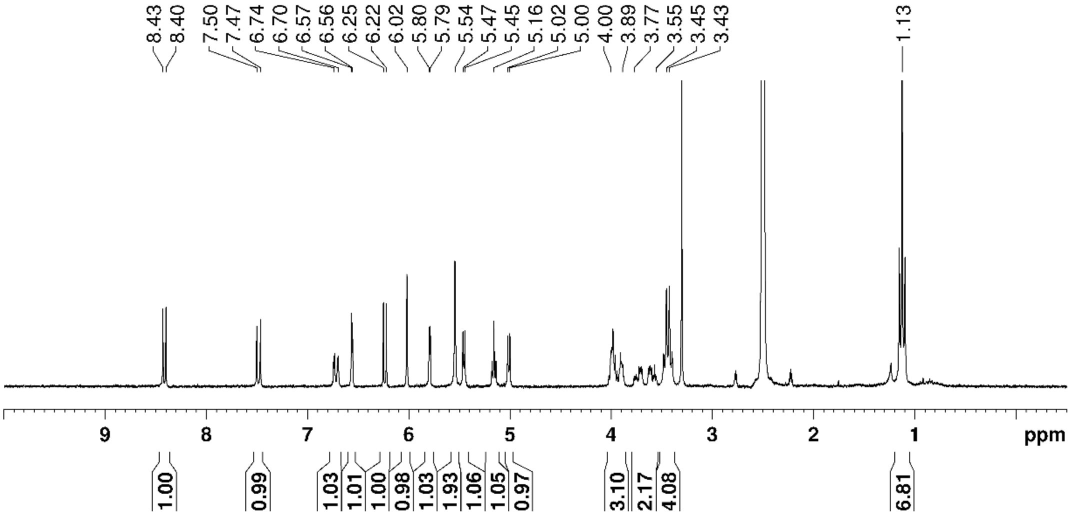

Figure S21. ^{1}H-NMR spectra of compound **3** recorded on a *Bruker DPX250* spectrometer in DMSO-d_6 (250 MHz, 298 K).

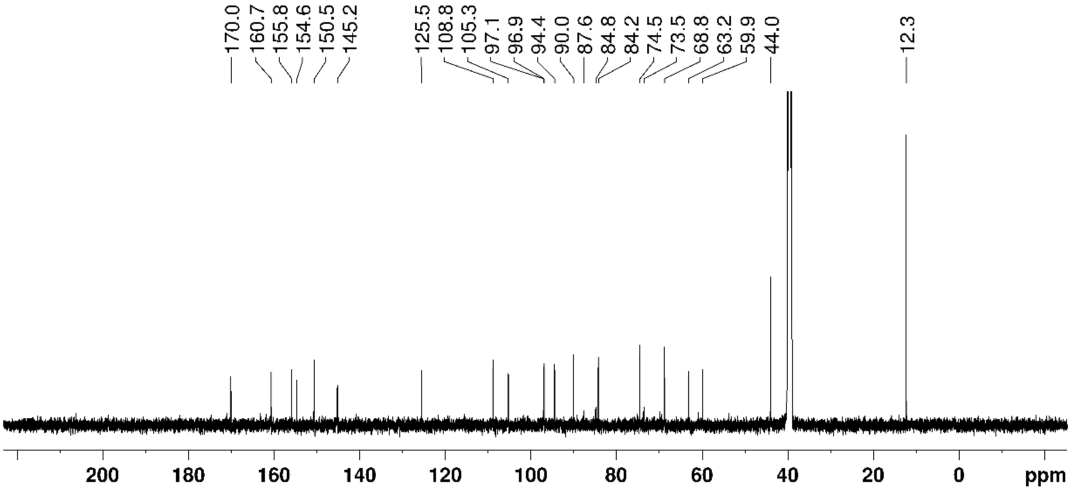

Figure S22. ^{13}C-NMR spectra of compound **3** recorded on a *Bruker AV500* spectrometer in DMSO-d_6 (126 MHz, 298 K).

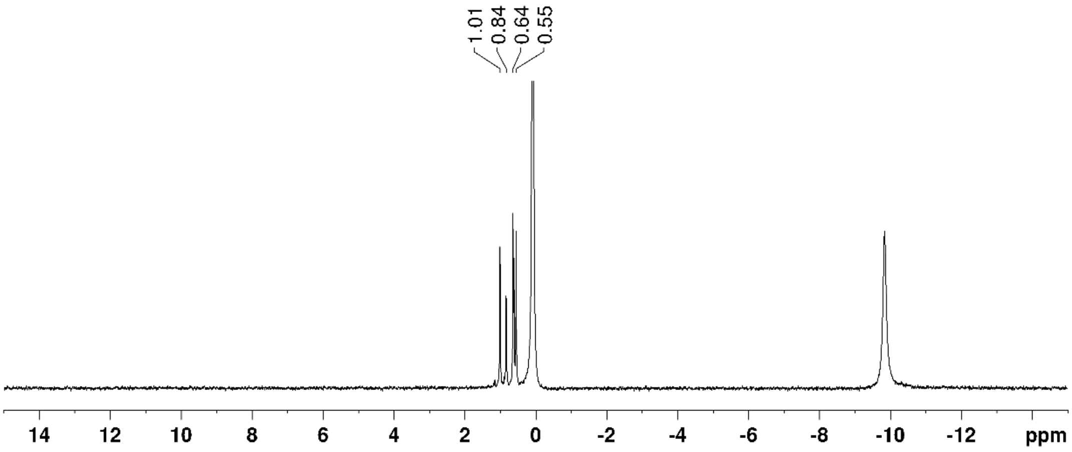

Figure S23. ^{31}P-NMR spectra of the crude product mixture of **3',5'-** and **2',5'-pU^{DEACM}p** recorded on a *Bruker AV500* spectrometer in D_2O (202 MHz, 298 K).

9.6. Test ligation of pU^{DEACM}p

The test ligation was performed using a mixture of 25 µM **Test-RNA1** and 100 µM 3',5'/2',5'-**pU^{DEACM}p** in a total volume of 50 µL containing 1x *T4* RNA ligase 1 buffer, 10% (v/v) DMSO and 2% *T4* Rnl1. The mixture was incubated at 37 °C overnight. The enzyme was heat deactivated 65 °C for 10 min. The reaction mixture was analyzed by RP-HPLC (gradient Table S2) and mass spectrometry. An aliquot was irradiated at 420 nm for 10 min and also RP-HPLC analyzed. Mass spectra after ligation and dephosphorylation can be seen in Figure S24 (upper part left and right). RP-HPLC chromatograms of the ligation and irradiation also can be seen in Figure S24 (lower part, left). After evaporation at 4 °C, the 3'-extended product was dephosphorylated using 2% (v/v) Shrimp Alkaline Phosphatse in *Cut Smart Buffer*. The mixture was incubated at 37 °C for 30 min and afterwards RP-HPLC-analyzed. The chromatogram of the dephosphorylation can be seen in Figure S24 (lower part, right). the incorporation of the newly synthesized DEACM-caged uridine bisphosphate in combination with correctly measured masses confirm the identity of the bisphosphate.

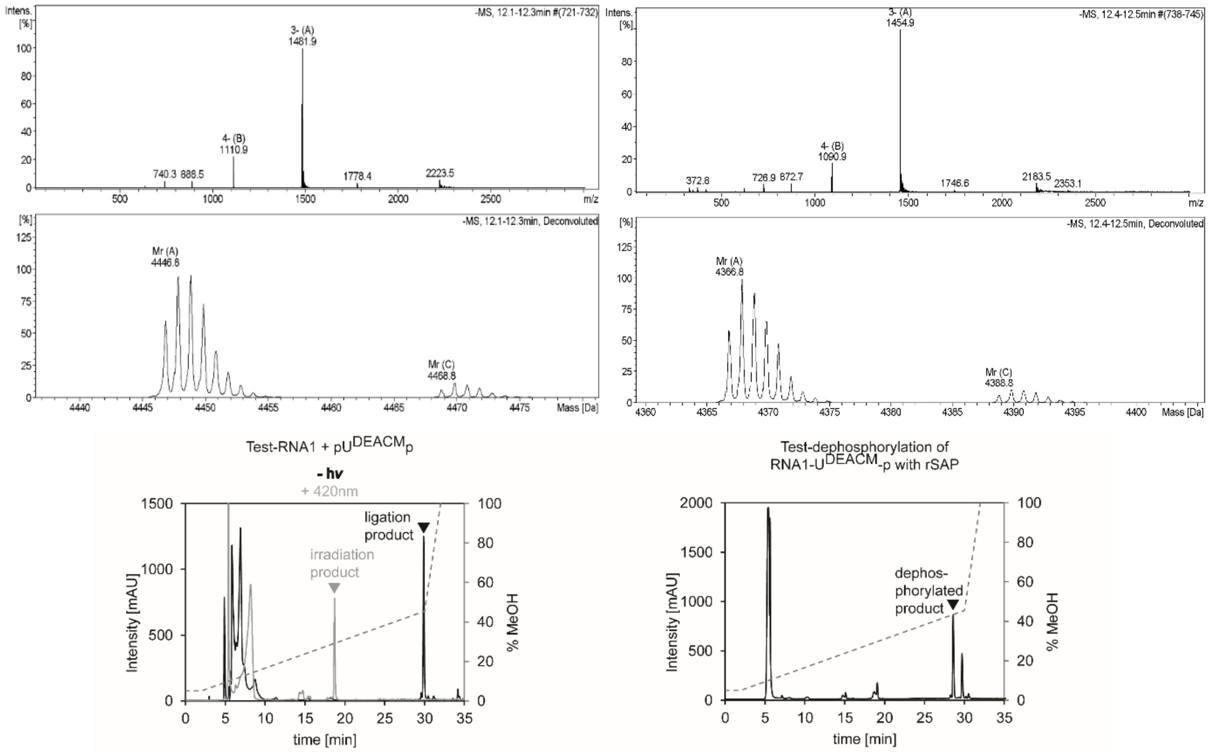

Figure S24. Upper part: Measured mass spectra of the tested 3'-extension of **Test-RNA1** and **pU^{DEACM}p** (left spectrum). The calculated mass for the lilgated product (**Test-RNA1-U^{DEACM}-p**) is 4447.7, the measured mass is 4446.8. The right spectrum represents the measured mass after 3'-dephosphorylation. The calculated mass for the dephosphorylated product (**Test-RNA1-U^{DEACM}-OH**) is 4367.7, the measured mass is 4366.8. Lower part: RP-HPLC chromatograms after ligation (left) and before (-h*v*, black lines) and after irradiation (+420 nm, grey line). A RP-HPLC chromatogram after dephosphorylation can be seen on the right side.

10. Test ligations with DNA/RNA chimera

We tested if the enzymatic incorporation of photocleavable protecting groups is still possible with DNA/RNA chimera. Mixmers are often used to reduce RNase activity. Therefore, we synthesized two DNA/RNA mixmers (see Section 16). Care was taken to ensure that the RNA to be elongated had at least three RNA bases at the 3'-end so that it could be recognized as an acceptor fragment by RNA ligases. The sequence was synthesized with a phosphodiester backbone as well as with a fully thiolated backbone. The 3'-extensions were performed in solution in total volumes of 100 µL using 0.3 µM **mixmer** and 0.9 µM **pC^{NPE}p** in 1x *T4* RNA ligase 1 buffer with 10% (v/v) Rnl1. The reaction mixtures were incubated at 37 °C/300 rpm for 8 h. The enzyme was heat deactivated at 65 °C for 10 min. The reaction mixtures were then purified via RP-HPLC (gradient Table S2) and confirmed by mass spectrometry. The solvent was removed using a vacuum concentrator. The 3'-extended mixmers were then 3'-dephosphorylated with rSAP and again analyzed by RP-HPLC and mass spectrometry. Sequences and calculated and measured masses are given **in Table S9**. Mass spectra recorded on a *Bruker micrOTOF-QII* device (left) and the RP-HPLC chromatogram of the purification (right) are shown below.

Table S9. Sample names, sequences and calculated and measured masses of the 3'-extended DNA/RNA mixmers.

Sample	Sequence	Exact calcd Mass [Da]	Measured Mass [Da]
**Sequence 6-C^{NPE}-p (phosphodiester)**	5'-d[CCC CCC CC]-r[C CCC **C^{NPE}**]-p-3'	4004.6	4005.1
**Sequence 7-C^{NPE}-p (phosphorothioate)**	5'-d[CCC CCC CC]-r[C CCC **C^{NPE}**]-p-3'	4180.4	4100.9
**Sequence 6-C^{NPE}-OH (phosphodiester)**	5'-d[CCC CCC CC]-r[C CCC **C^{NPE}**]-OH-3'	3924.7	3925.2
**Sequence 7-C^{NPE}-OH (phosphorothioate)**	5'-d[CCC CCC CC]-r[C CCC **C^{NPE}**]-OH-3'	4100.4	4100.9

Sequence 6 (phosphodiester backbone) + pC^{NPE}p

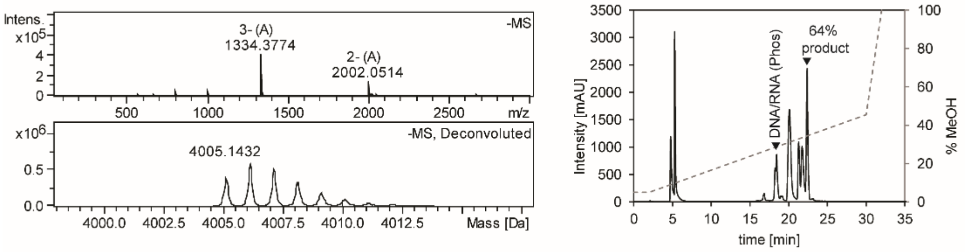

Sequence 7 (phosphorothioate backbone) + pC^{NPE}p

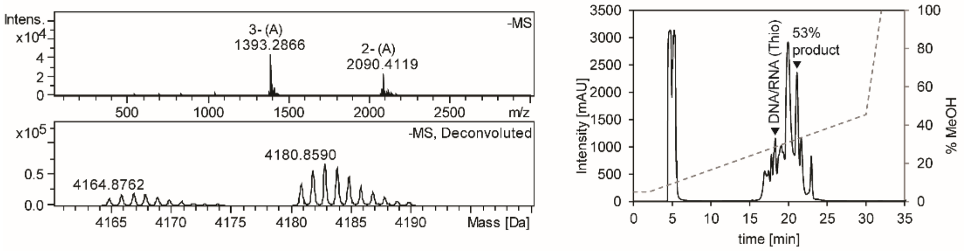

Sequence 6-C^{NPE}-p (phosphodiester backbone) + rSAP

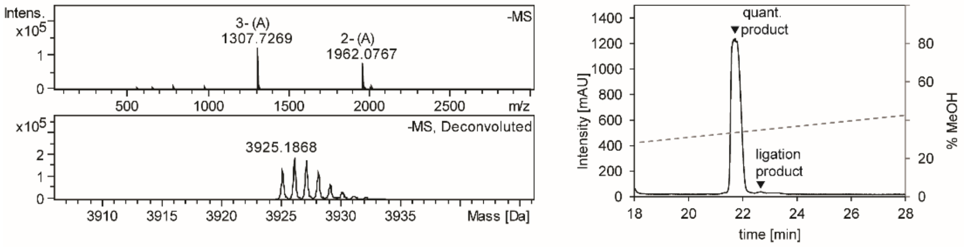

Sequence 7-C^{NPE}-p (phosphorothioate backbone) + rSAP

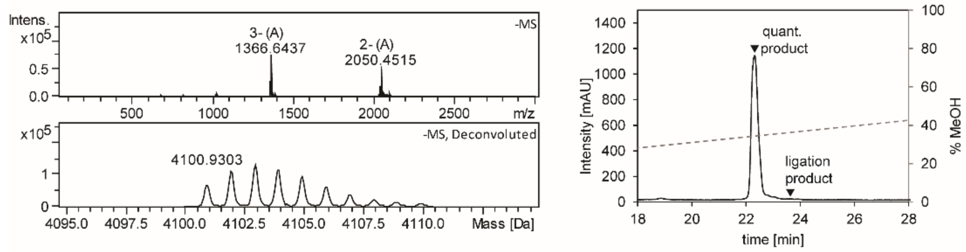

To achieve an internal NPE-modification, the purified DNA/RNA-C^{NPE} mixmers were splint ligated in solution using *T4* Rnl2. Reaction mixtures with a 1:1:1 ratio of **mixmer-C^{NPE}**, **5'p-RNA2** and **DNA splint** (each 8 µM) in 1x *T4* DNA ligase buffer and 5% (v/v) *T4* Rnl2 were incubated at 37 °C/300 rpm for 3 h. The DNA splint was digested with *TURBO*^{TM} *DNase* was from *Thermo Fisher* using the manufacturers protocol. In Figure S25 a denaturing polyacrylamide gel with compared splinted ligations can be seen. Yields were determined by analyzing the band intensities using *Image Lab*^{TM}.

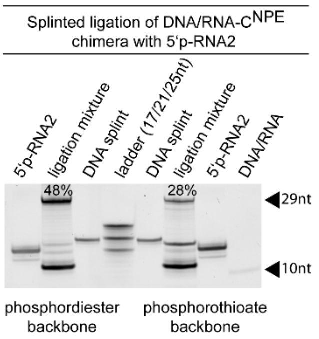

Figure S25. 20% denaturing polyacrylamide gel presenting the compared splinted ligation of a **DNA/RNA-C^{NPE}** mixmer with phosphodiester backbone and **5'p-RNA2** and a **DNA/RNA-C^{NPE}** mixmer with phosphorothioate backbone and **5'p-RNA2**. Lane 1: 5'-phosphorylated RNA2; lane 2: ligation mixture of a modified phosphodiester mixmer; lane 3: DNA splint; lane 4: RNA ladder (17, 21, 25 nt) as reference; lane 5: DNA splint; lane 6: ligation mixture of a modified phosphorothioate mixmer; lane 6: 5'-phosphorylated RNA2; lane 7: unmodified DNA/RNA mixmer with a phosphorothioate backbone.

11. Test ligations with 2'OMe substituted RNA

Test 3'-extension of a fully 2'OMe modified RNA was performed in solution in a total volume of 50 µL using 50 µM **2'OMe-RNA (Sequence 8)** and 150 µM **pC^{NPE}p** in 1x *T4* RNA ligase 1 buffer with 10% (v/v) DMSO and 10% (v/v) *T4* Rnl1. The reaction mixture was incubated at 37 °C/300 rpm for 3 h. After heat deactivation of the enzyme at 65 °C for 10 min, the reaction mixture was analyzed via RP-HPLC and a denaturing polyacrylamide gel. No product formation could be observed. An analysis via mass spectrometry confirmed this result. In Figure S26 the denaturing polyacrylamide gel is shown.

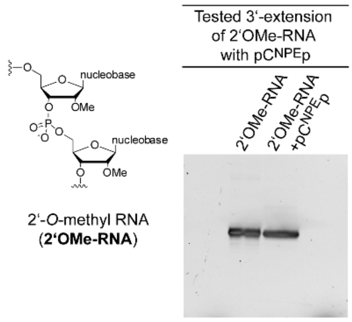

Figure S26. 20% denaturing polyacrylamide gel of the tested enzymatic 3'-extension of a fully 2'OMe-modified RNA. No product could be identified.

12. Tested sodium periodate capping and 3'-modification with a rhodamine derivative

The product of an enzymatic 3'-extension has a 3'-terminal phosphate group. This phosphate serves as a kind of protecting group that prevents the multiple incorporations of modified bisphosphates. However, since most 3'-extensions using *T4* RNA ligase 1 are not quantitative, a small amount of starting material with a 3'-hydroxyl group remains in the ligation mixture. To avoid the further extension of unmodified starting material a sodium periodate capping step can be performed resulting in a 2',3'-dialdehyde. Buffer solutions should be carefully removed during washing steps because ATP and DTT would disturb the periodate oxidation. The capping reaction should also be performed in a dark reaction tube because of the light sensitivity of $NaIO_4$. We tested if a test-RNA could be 3'-labeled with a rhodamine via morpholino chemistry after an oxidation step. The procedure was performed inspired by literature procedure.[4] Care was taken to ensure that the reactions were carried out under mild conditions and within short reaction times to prevent degradation of the RNA and dissociation of the biotin/streptavidin interaction. For this test, we performed a sodium periodate capping reaction in a total volume of 100 µL. 10 µM of an **RNA-34mer (Test-RNA2)** were bound to freshly prepared streptavidin beads in deionized water and 15 mM $NaIO_4$ were incubated at 37 °C/300 rpm under light exclusion for 15 min. In a second reaction tube 67 mg of the used rhodamine derivative (132 µmol, 1 eq) were dissolved in 0.6 mL of a 9:1 mixture of H_2O/MeOH. 26 mg boric acid (418 µmol, 3.8 eq) were added to the dissolved rhodamine. After 15 min, 30 µL of the prepared rhodamine/boric acid mixture were added to the capping reaction mixture. The pH was carefully adjusted with Et_3N to 8.5. The mixture was incubated at 37 °C/500 rpm for 1.0 h. Subsequently 1.5 µL of a 5 M sodium cyanoborohydride solution (in NaOH) were added. The reaction mixture was incubated at 37 °C/500 rpm for 45 min. During the whole reaction pathway, the suspension was pink colored, a color that comes from the rhodamine derivative but we were also able to identify partially dissociated RNA. For this reason, the reaction times were kept short. The reaction

was stopped by washing the beads five times with binding buffer. In the first washing step, the supernatant was pink colored. During the washing steps, the supernatant lost color, but the beads remained dark purple. After the last washing step, the beads were suspended in 100 µL deionized water and heated to 75 °C for 10 min to release the RNA. The suspension turned into a pink color. The beads were then pulled to the wall of the reaction tube using a magnet and the supernatant was analyzed by RP-HPLC and mass spectrometry. In Figure S27 an overview of the reaction as well as measured mass spectra and a RP-HPLC chromatogram can be seen. The measured mass fits perfectly with the calculated mass of the product in presence of sodium [M+Na$^+$] (calcd: 11807.8, measured: 11807.2). The released RNA appears as a pink solution, a typical color in combination with a rhodamine.

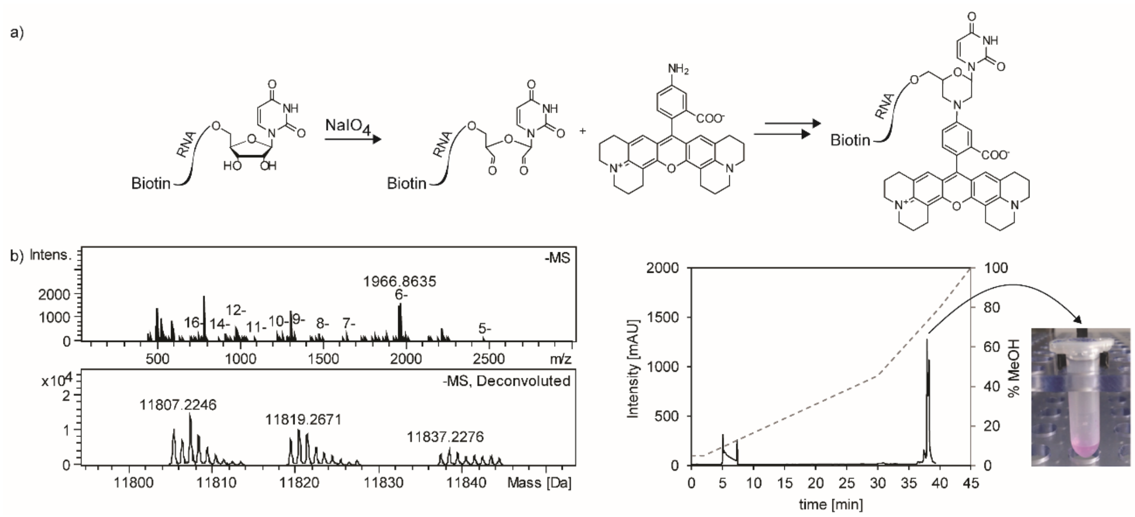

Figure S27. Summary of the tested 3'-labeling reaction. a) Schematic representation of the reaction pathway. After a sodium periodate capping of the 3'-end, the dialdehyde was labeled with a rhodamine derivative (synthesized by Rebekka Weber, *Heckel Group*). b) Measured mass spectrum of the purified RP-HPLC product. The calculated mass as sodium adduct is 11807.8, the mass found as adduct of the product with sodium is 11807.2. Also the RP-HPLC purified RNA appears as pink colored solution.

13. Test ligations with a photocleavable biotin residue and p(*m*Azo)p

In case of the incorporation of photoswitchable residues like azobenzenes, the biotin residue can be introduced in combination with a photocleavable (NPE) linker. Upon irradiation at 365 nm, this linker can be cleaved, releasing an 5'-phosphorylated RNA with photoswitchable modifications instead of a attached 5'-biotin residue. For this, RNA1 was synthesized with a 5'-photocleavable biotinylated residue **Bio-PC-RNA1** via chemical solid-phase synthesis (see Section 16). This **Bio-PC-RNA1** was 3'-extended with 3',5'-*m*-azobenzene bisphosphate. The reaction was performed using 50 µM **Bio-PC-RNA1** and 150 µM **p(*m*Azo)p** in 1x *T4* RNA ligase buffer 1 with 10% (v/v) DMSO and 10% (v/v) *T4* Rnl1. The reaction mixture was incubated at 37 °C/300 rpm for 3 h. After heat deactivation at 65 °C for 10 min, an aliquot was analyzed via RP-HPLC and mass spectrometry. 10% (v/v) *Cut Smart buffer* and 10% (v/v) rSAP was added to the remaining reaction mixture. For dephosphorylation, the reaction mixture was incubated again at 37 °C/300 rpm for 2 h. Again, an aliquot was analyzed by RP-HPLC and mass spectrometry using the same conditions as before. Sequences and calculated and measured masses are given in Table S10.

Table S10. Sample name, sequence and calculated and measured masses of the 3'-extension of a photocleavable biotinylated RNA after dephosphorylation.

Sample	Sequence	Exact calcd Mass [Da]	Measured Mass [Da]
**Bio-PC-RNA1-*m*Azo-p**	5'-**Bio-(PC linker)**-r[GGC UAC GUA G***m*****Azo**]-OH-3'	4402.8	4403.3

Bio-PC-RNA1 + p(*m*Azo)p + rSAP

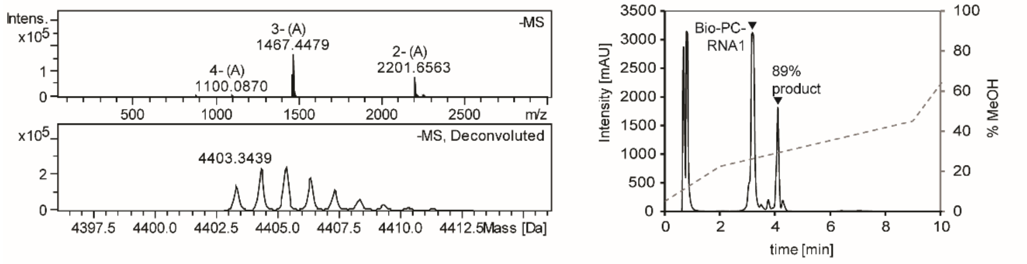

14. Test ligations with an NPE-cleavable 5’,3’-diphosphorylated dinucleotide

The incorporation of photolabile protecting groups within the phosphodiester backbone of oligonucleotides gives the possibility of light induced strand breaks. Up to now, it was not clear if a modification within the phosphodiester backbone would be accepted and incorporated by the ligases used in this study. Therefore, we designed and synthesized (see Section 16) a photocleavable dinucleotide which is the smallest possible unit bearing a photolabile protecting group in the backbone. This **‘photocleavable strand break unit’ (PU)** unit is formed of an NPE linked cytidine and uridine dinucleotide (**p(C-PC linker-U)p**) (see Figure S28). The streptavidin bound 3’-extension was performed using 25 µM **Bio-RNA1** and 75 µM of the **photocleavable strand break unit** in a total volume of 50 µL. 5% (v/v) *T4* Rnl1 were added and the reaction mixture was incubated at 37 °C/300 rpm for 4 h. An Aliquot was analyzed by RP-HPLC (gradient Table S2) and mass spectrometry. A buffer exchange was performed and the extended RNA was 3’-dephosphorylated with rSAP at 37 °C/300 rpm for 3 h. Again, an aliquot was analyzed by RP-HPLC and mass spectrometry using the same conditions as before. Sequences and calculated and measured masses are given in Table S11. Mass spectra recorded on a *Bruker micrOTOF-QII* device (left) and the RP-HPLC chromatogram of the purification (right) are shown below.

Table S11. Sample names, sequences and calculated and measured masses of the incorporation of a photocleavable strand break unit.

Sample	Sequence	Exact calcd Mass [Da]	Measured Mass [Da]
**Bio-RNA1-PU-p**	5’-**Bio**-r[GGC UAC GUA G**C-PC linker-U**]-p-3’	4587.7	4586.3
**Bio-RNA1-PU-OH**	5’-**Bio**-r[GGC UAC GUA G**C-PC linker-U**]-OH-3’	4507.7	4506.4
**Bio-RNA1-PU-RNA2**	5’-**Bio**-r[GGC UAC GUA G**C-PC linker-U** UCA GUU GGU UAG AGC ACA]-OH-3’OH-3’	10327.5	10328.4

Bio-RNA1 + p(PU)p

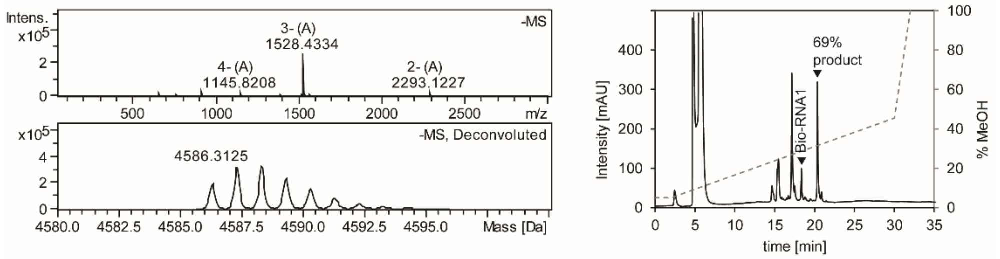

Bio-RNA1-PU-p + rSAP

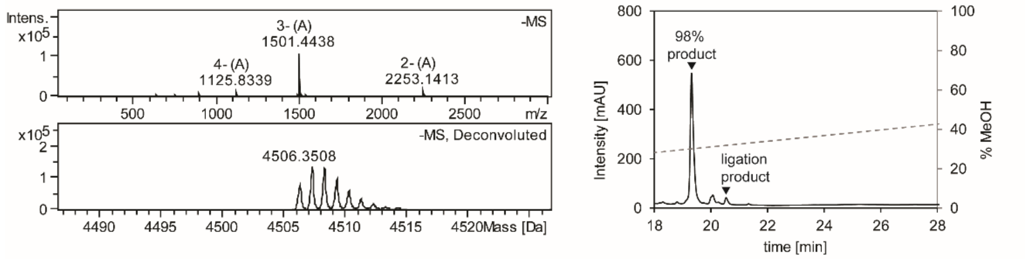

A splinted ligation with the backbone modified RNA fragment was also tested. DNA splint 1+2 was used, although this was one base too short and therefore not a perfect matching splint. To compensate this fact, the ligation temperature was lowered to 25 °C and the ligation time was extended to 6 h. A 1:1 mixture of **5’-p RNA2** and **DNA splint** in *T4* DNA ligase buffer was heated to 80 °C/300 rpm for 4 min and then cooled to 37 °C/300 rpm to pre-anneal both oligonucleotides. This mixture was then transferred to an aliquot of **Bio-RNA1-PU**. 10% (v/v) of *T4* Rnl2 were added and the mixture incubated at 25 °C/300 rpm. After a subsequent DNase digestion the ligation mixture was analyzed via denaturing polyacrylamide gel (see Figure S28b) and RP-HPLC (Figure S28c). Figure S28d shows the mass spectrum of the ligation product. The structure of the incorporated photocleavable dinucleotide is also shown in Figure S28a.

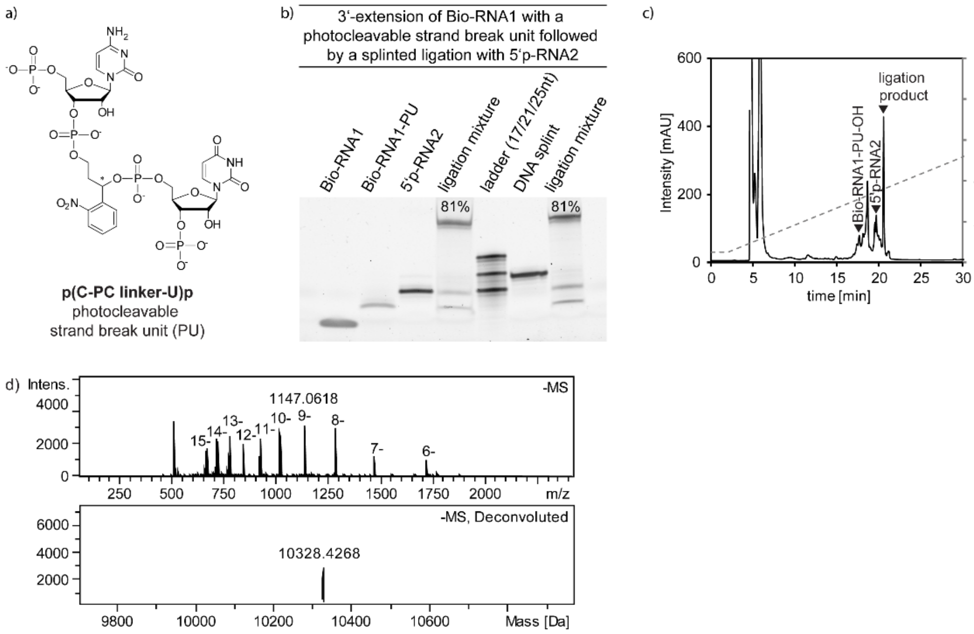

Figure S28. a) Structure of the photocleavable dinucleotide with NPE-photocage in the phosphodiester backbone. b) 20% denaturing polyacrylamide gel representing the enzymatic incorporation of the dinucleotide. Lane 1: starting RNA fragment **Bio-RNA1**; lane 2: RP-HPLC purified 3'-extended and dephosphorylated product **Bio-RNA1-PCunit**; lane 3: **5'p-RNA2**; lane 4: ligation mixture after 6 h reaction time; lane 5: RNA ladder (17, 21, 25 nt) as reference; lane 6: DNA splint; lane 7: again the ligation mixture after 6 h reaction time. Yields were determined by comparing the band intensities of the starting material and the product using *Image Lab*TM. c) RP-HPLC chromatogram after the splinted ligation. The product is eluted after 20.57 min. d) Mass spectrum of the ligation product.

15. Summarized Ligation Yields

We could show in various applications, that an incorporation of modified nucleotide bisphosphates into different kinds of oligonucleotides is possible. Figure S29 shows a summary of achieved yields.

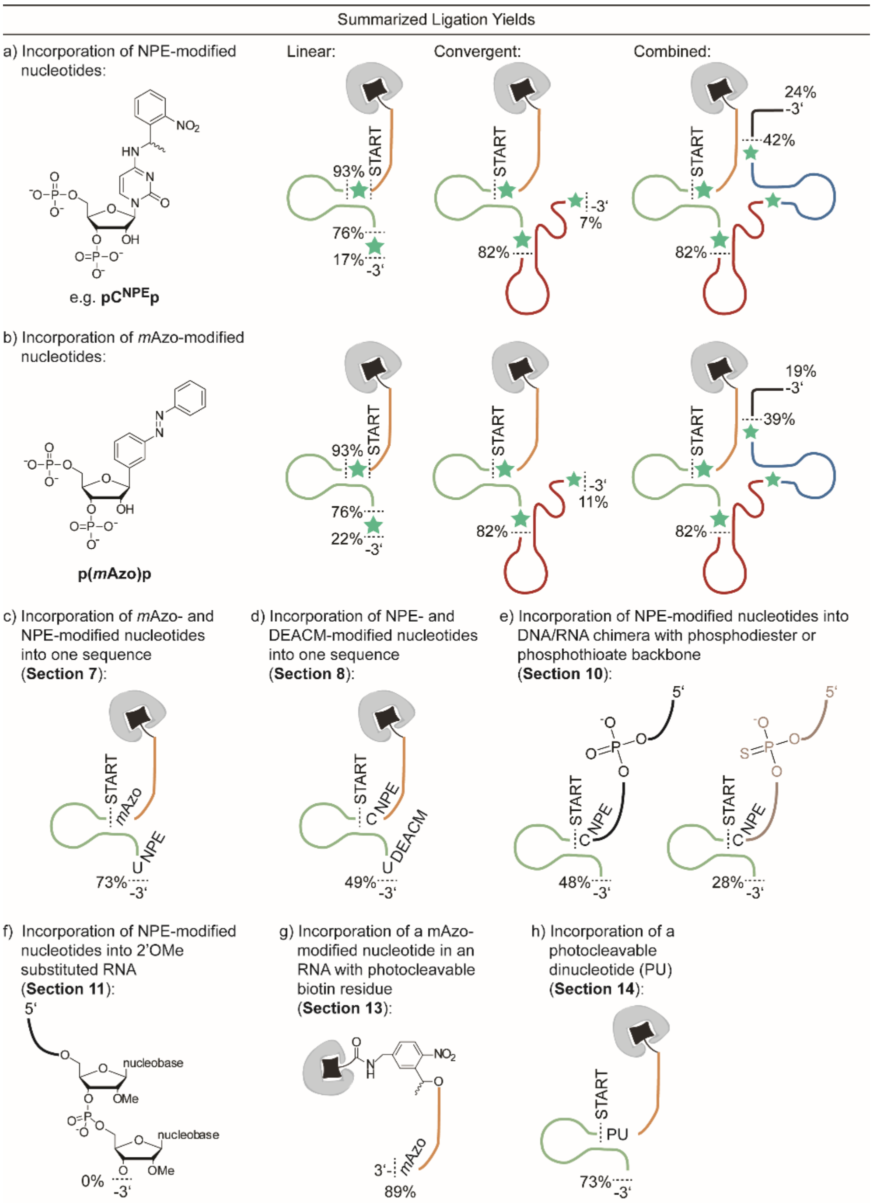

Figure S29. Overview of the ligation yields.

16. Chemical Solid-Phase Synthesis

Chemical oligonucleotide syntheses were either performed in 1 µmol scales on an *ABI392 DNA/RNA synthesizer* from *Applied Bioscience* or in 17 µmol scales on an *Oligopilot OP10 plus* from *GE Healthcare*. Commercially available phosphoramidites, CPG materials, and reagents were purchased from *Glen Research, LGC Link, emp BIOTECH,* or *Sigma Aldrich.* As activator 0.3M BTT in acetonitrile was used. Pac_2O was used as capping reagent. All unmodified oligonucleotides were synthesized and deprotected under standard conditions, while the syntheses of 5'-phosphorylated or photoactivatable oligonucleotides were performed under *UltraMILD* conditions. The coupling time for DNA was 6 min. The coupling time for RNA or modified phosphoramidites was extended to 12 min. All sequences were synthesized DMTr-off. To remove the cyanoethyl groups, the CPG material was first flushed with 20% diethylamine in MeCN for 10 minutes, then washed with pure MeCN and dried under vacuum. The further deprotection was performed according to protocols from *Glen Research*[5].

DNA Deprotection: For deprotection, the solid-phase material was treated with ammonium hydroxide solution (32%) for 4 h at 60 °C. The solvent was removed at 4 °C under vacuum using a *Concentrator plus* from *Eppendorf*.

RNA Deprotection: For standard deprotection, the solid-phase material was treated with a 3:1 mixture of ammonium hydroxide solution (32%) and EtOH for 4 h at 60 °C. For *UltraMILD* deprotection of modified RNA, the cleavage time was reduced to 2 h at 60 °C. The solvent was removed at 4 °C using a vacuum concentrator. For the removal of the 2'-TBDMS protecting groups, the residue was dissolved in 115 µL DMSO. 60 µL Et_3N and 75 µL $Et_3N·3HF$ were added and the mixture was incubated for 2.5 h at 60 °C. For precipitation, 10% (v/v) of 3M NaOAc and 4 times the volume of prechilled *n*-butanol were added to the cooled mixture. The fully deprotected RNA was precipitated 2.5 h at -20 °C and pelleted by centrifugation for 30 min at 4 °C. The precipitation was repeated at least 2 times to remove all residual fluoride salts.

For the purification of all resulting oligonucleotides, all used aqueous buffer solutions were prepared with Milli-Q water, which was treated with 0.1% (v/v) diethyl pyrocarbonate overnight and autoclaved before usage. All resulting crude oligonucleotides were purified by RP-HPLC performed on an *Agilent 1200 series* instrument equipped with an *XBridge Peptide BEH C18 OBD Prep Column* (300 Å, 5 µm, 10x250 mm) or an *XBridge BEH C18 OBD Prep Column* (130 Å, 5 µm, 10x50 mm) from *Waters*. Elution was performed with 4.0 mL/min at 60 °C (solvent A: MeOH, solvent B: 400 mM HFIP 16.3 mM Et_3N, pH 8.0). Unmodified 5'OH-RNA fragments were solid-phase synthesized and also obtained from *Biomers*. Sequences, analytical RP-HPLC chromatograms, and measured mass spectra are shown in the next section.

16.1.Synthesized Sequences

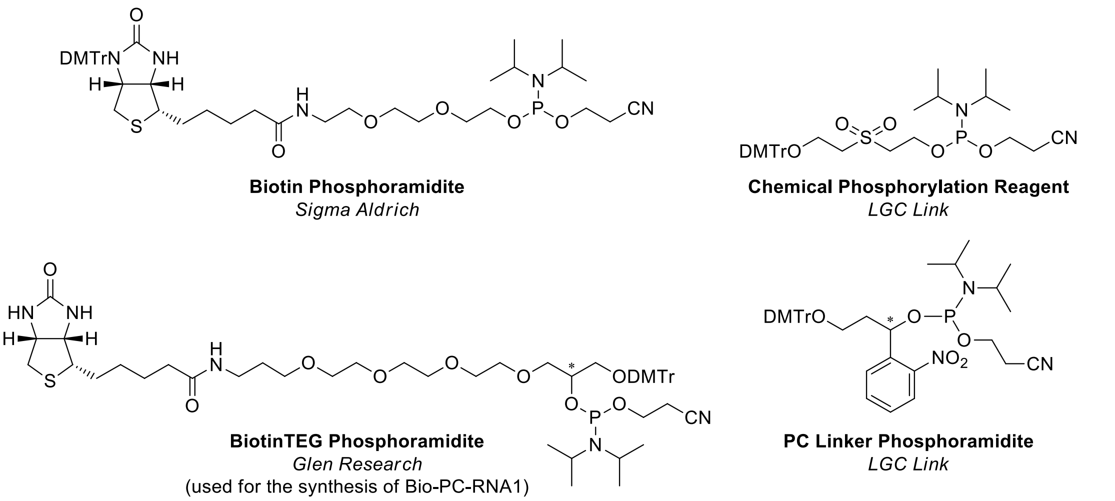

Figure S30. Structures and vendors of the non-standard phosphoramidites used. Biotinylated RNAs were typically synthesized using the biotin phosphoramidite from *Sigma Aldrich* only **Bio-PC-RNA1** was synthesized with the BiotinTEG phosphoramidite.

Table S12. Sample names, corresponding sequences, calculated and measured masses of all solid-phase synthesized sequences.

Sample	Sequence	Exact calcd Mass [Da]	Measured Mass [Da]
**5'OH-RNA1**	5'-OH-r[GGC UAC GUA G]-3'	3198.5	3198.5
**5'Bio-RNA1**	5'-**Bio**-r[GGC UAC GUA G]-3'	3636.6	3635.7
**5'Bio-PC-RNA1**	5'-**Bio-PC**-r[GGC UAC GUA G]-3'	4026.7	4027.2
**5'p-RNA2**	5'-p-r[UCA GUU GGU UAG AGC ACA]-3'	5833.8	5834.5
**5'p-RNA3**	5'-p-r[CAC UCA UAA UGA UGG GGU CA]-3'	6467.9	6468.6
**5'p-RNA4**	5'-p-r[AGG UUC GAA UCC CGU C]-3'	5134.0	5135.4
**5'p-RNA5**	5'-p-r[UAG CCA CCA]-3'	2876.4	2876.8
**5'OH-RNA2**	5'-OH-r[UCA GUU GGU UAG AGC ACA]-3'	5753.8	5754.5
**5'OH-RNA3**	5'-OH-r[CAC UCA UAA UGA UGG GGU CA]-3'	6387.9	6388.7
**5'OH-RNA4**	5'-OH-r[AGG UUC GAA UCC CGU C]-3'	5054.7	5055.4
**5'OH-RNA3^{NPE}-RNA4**	5'-OH-r[CAC UCA UAA UGA UGG GGU CA**C^{NPE}** AGG UUC GAA UCC CGU C]-3'	11958.6	11959.2

**5’OH-RNA3azo-RNA4**	5’-OH-r[CAC UCA UAA UGA UGG GGU CA(***m*****Azo)** AGG UUC GAA UCC CGU C]-3’	11864.6	11868.1
**DNA1 (Splint 1+2)**	5’-d[TCT AAC CAA CTG ATC TAC GTA GCC]-3’	7253.3	7253.3
**DNA2 (Splint 2+3)**	5’-d[CCA TCA TTA TGA GTG TTG TGC TCT AAC CAA C]-3’	9440.6	9440.6
**DNA3 (Splint 3+4)**	5’-d[GAT TCG AAC CTG TGA CCC CAT CA]-3’	6965.2	6965.4
**DNA4 (Splint 4+5)**	5’-d[GGT GGC TAC GAC GGG ATT CGA A]-3’	6837.2	6837.9
**Sequence 6 (phosphodiester)**	5’-d[CCC CCC CC]-r[C CCC]-3’	3470.6	3471.0
**Sequence 7 (phosphorothioate)**	5’-d[CCC CCC CC]-r[C CCC]-3’	3646.3	3646.8
**Photocleavable Strand Break Unit (PU)**	5’-p-r[C**(PC linker)**U]-P-3’	967.1	967.2
**Sequence 8**	5’-OH-2’OMe-r[AGC UUG AGA UAA GUC CGA CGA UCC U]-3’ synthesized by Janik Kaufmann (*Heckel Group*)	8328.5	8328.6
**Test-RNA1**	5’-OH-r[GGA UAG UGA UCC]-3’	3835.3	3834.0
**Bio-Test-RNA2**	5‘-**Bio**-r[GGC UAC GUA GCU CAG UUG GUU AGA GCA CAU CAC U]-3'	11308.6	11308.8

16.2.Mass spectra and analytical RP-HPLC chromatograms

The purity of all synthesized oligonucleotides was confirmed by analytical RP-HPLC (right) and HPLC-MS (left). RP-HPLC was performed on an *Agilent 1200 series* equipped with an *XBridge Peptide BEH C18* column (300 Å, 3.5 µm, 4.6x250 mm, 0.7 mL/min, 60 °C, solvent A: MeOH, solvent B: 400 mM HFIP 16.3 mM Et_3N, detected at 254 nm, Table S2) from *Waters*. Uridine was used as an internal standard (elution after 5 min). Mass spectra of all oligonucleotides were recorded on a *Bruker* micrOTOF-QII device (left: ESI, top: full-spectrum, bottom: deconvoluted molecular peak).

5’OH-RNA1

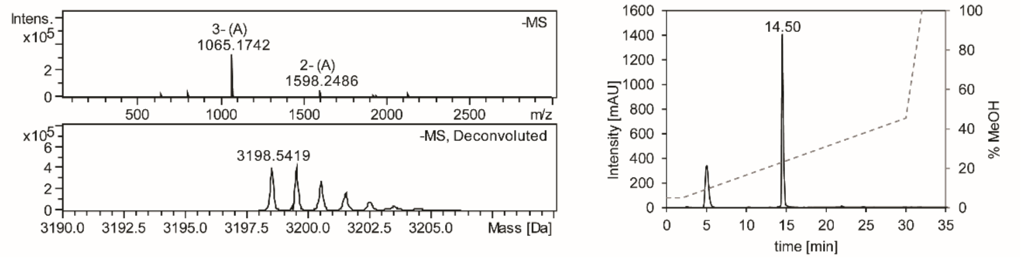

5’Bio-RNA1

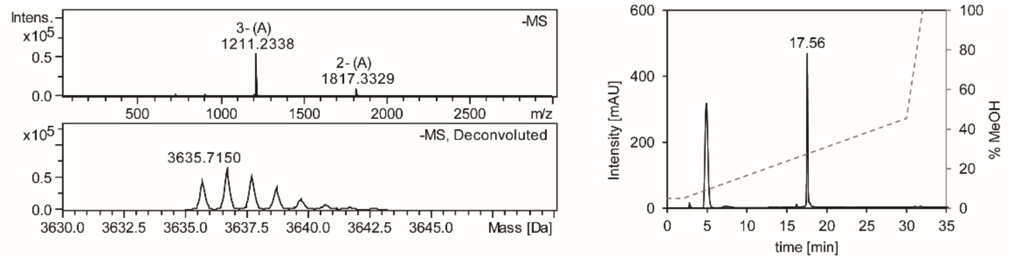

5’Bio-PC-RNA1

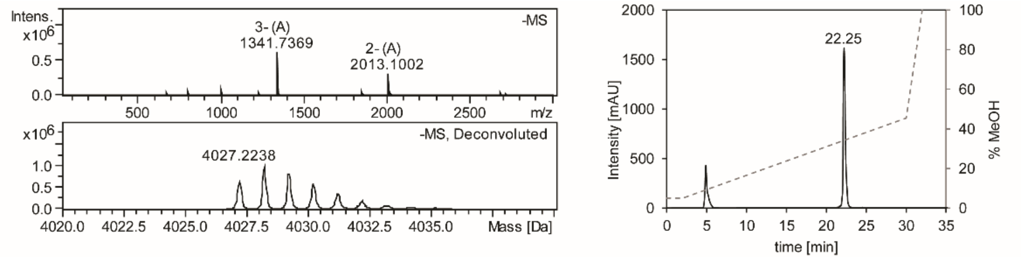

5’p-RNA2

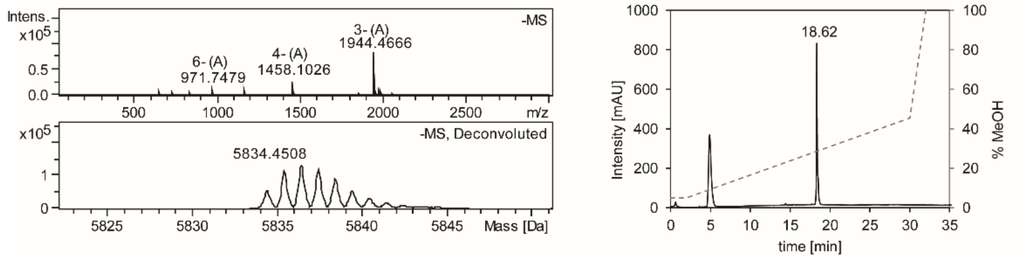

5’p-RNA3

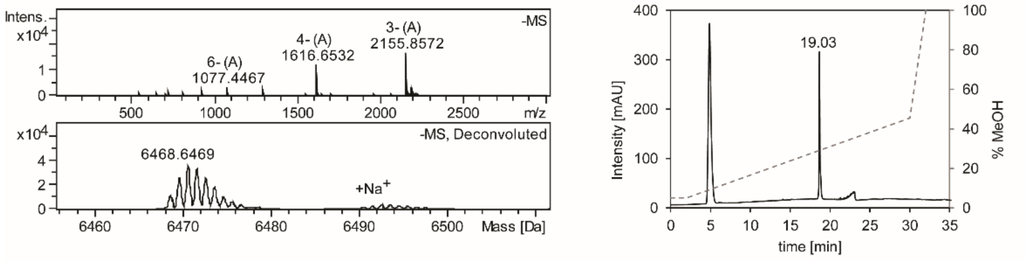

5’p-RNA4

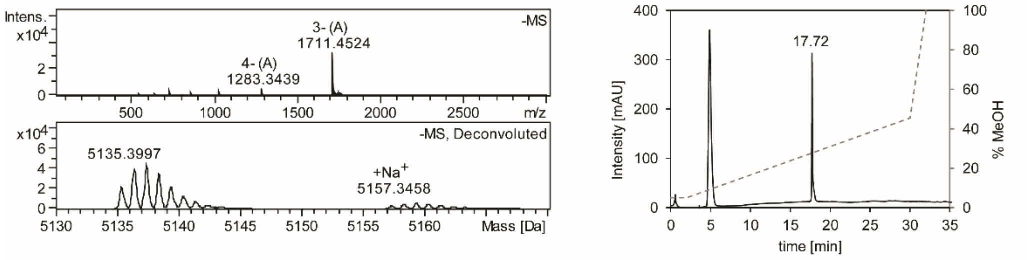

5’p-RNA5

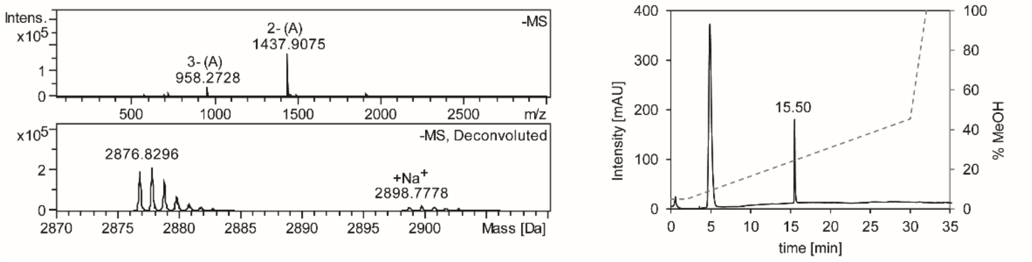

5’OH-RNA2

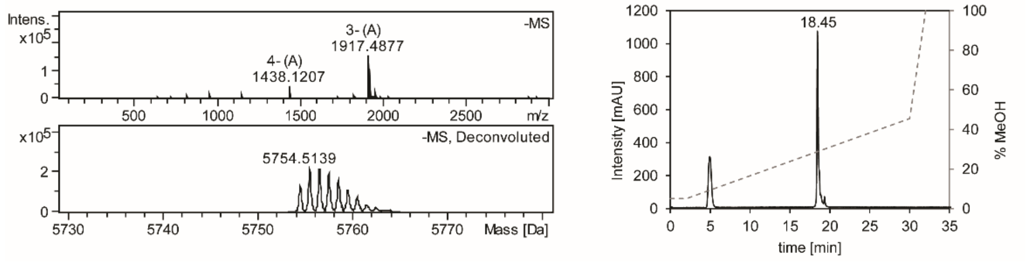

5’OH-RNA3

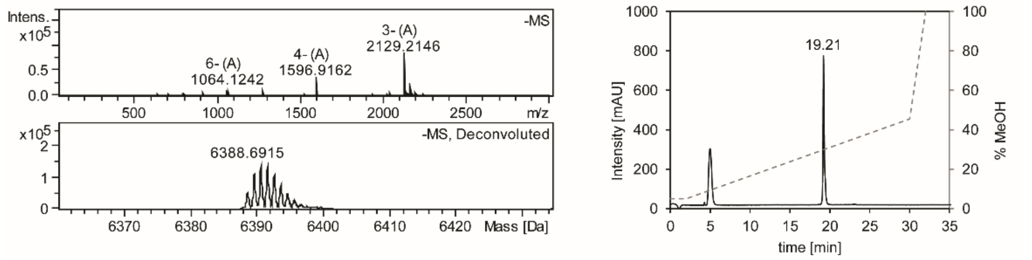

5'OH-RNA4

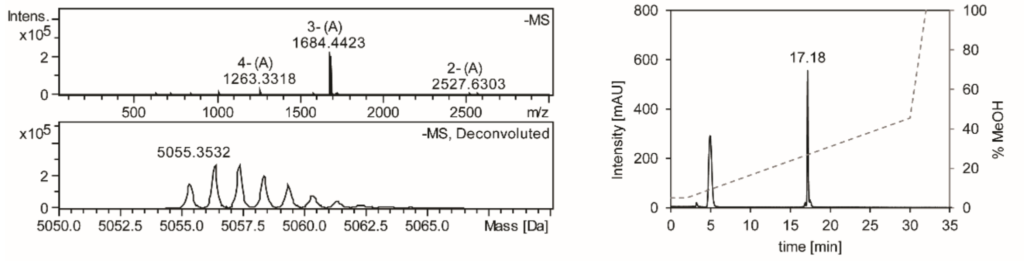

5'OH-RNA3^{NPE}-RNA4

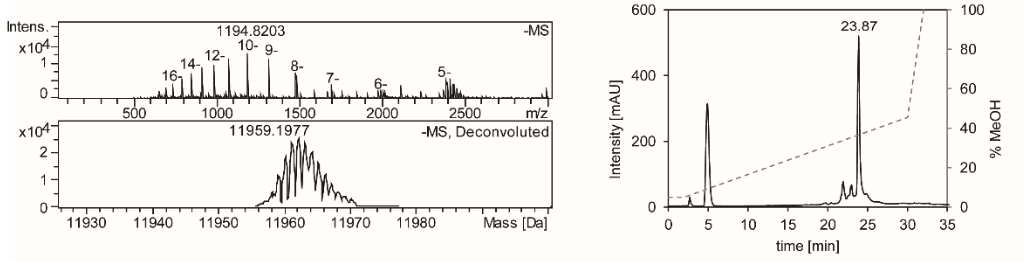

5'OH-RNA3azo-RNA4

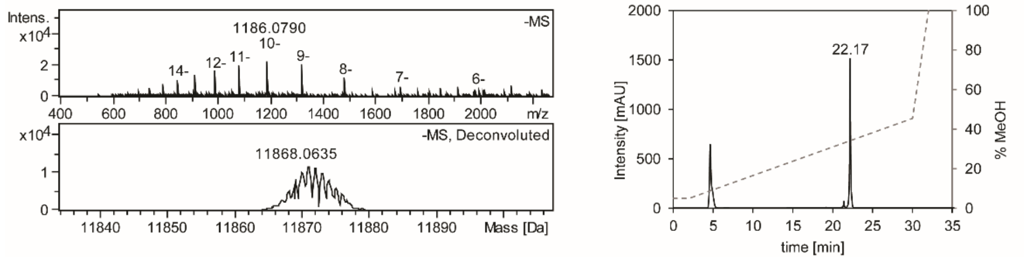

DNA1 (Splint 1+2)

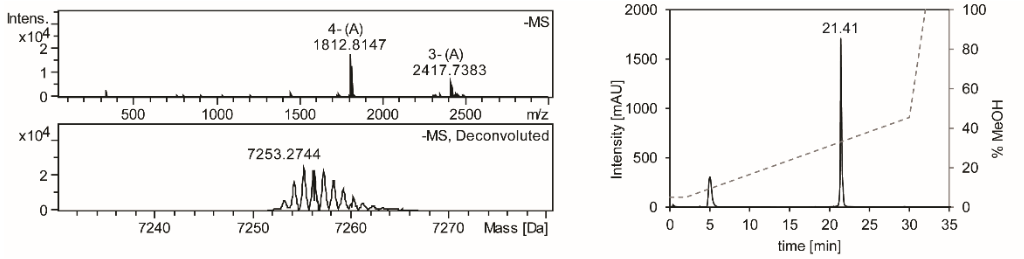

DNA2 (Splint 2+3)

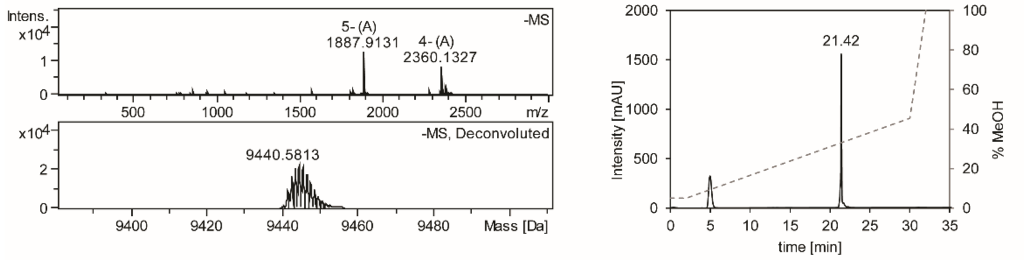

DNA3 (Splint 3+4)

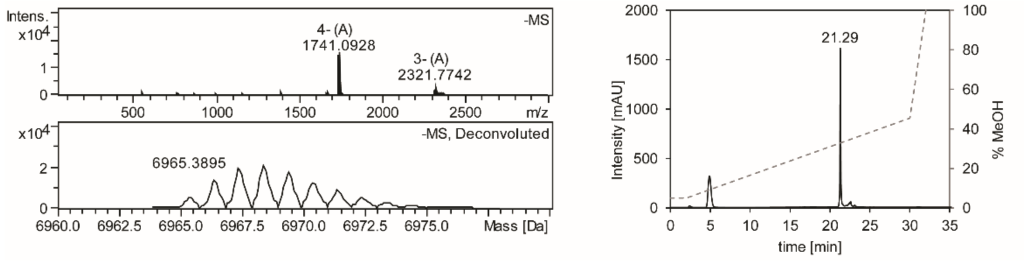

DNA4 (Splint 4+5)

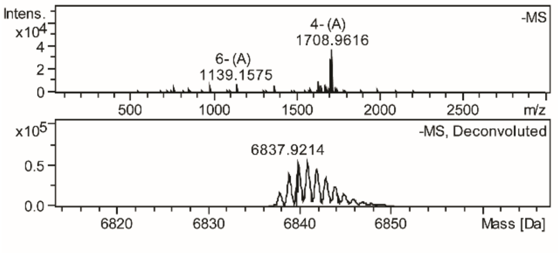

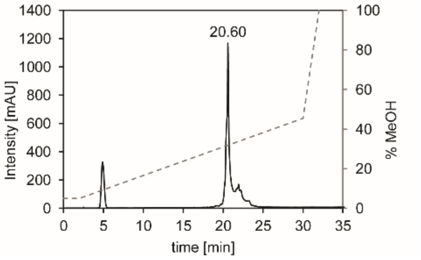

Sequence 6 (phosphodiester)

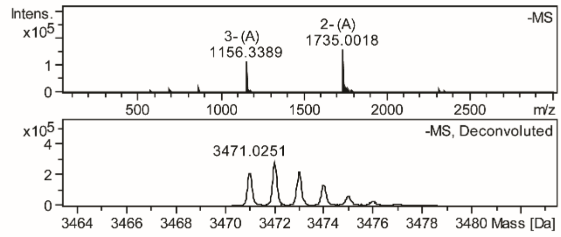

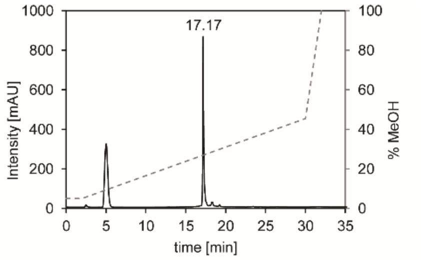

Sequence 7 (phosphorothioate)

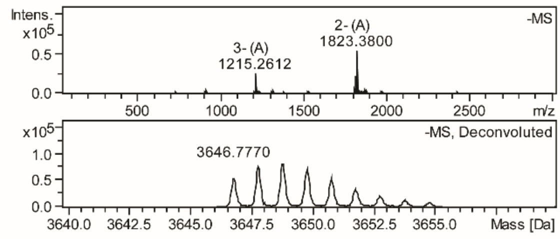

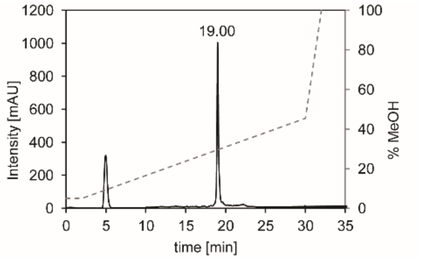

Photocleavable Strand Break Unit (PU)

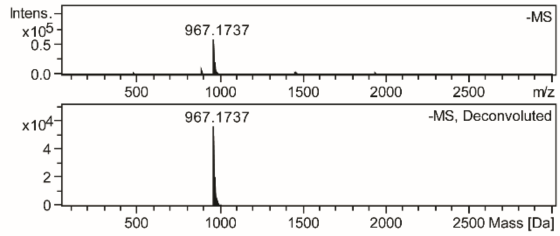

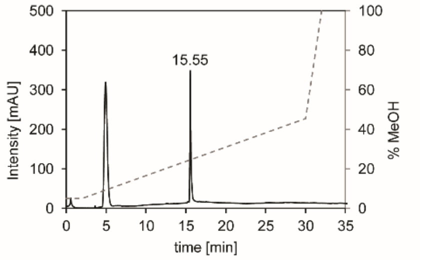

Sequence 8 (2'OMe RNA)

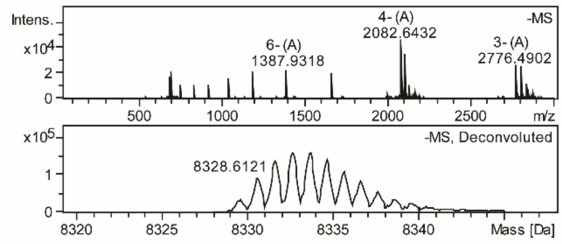

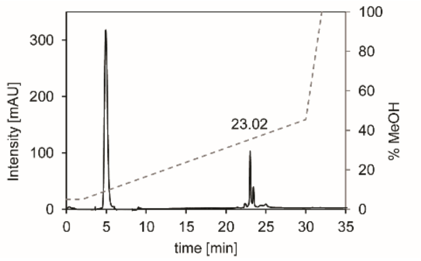

Test-RNA1

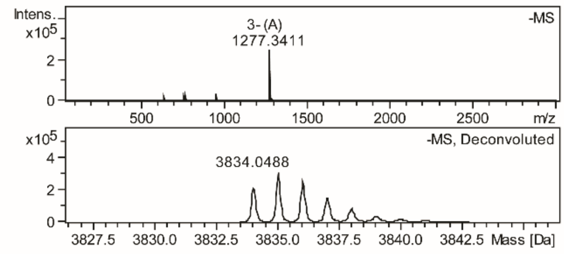

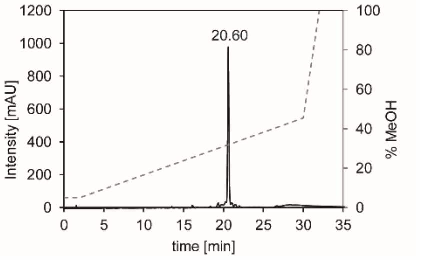

Bio-Test-RNA2

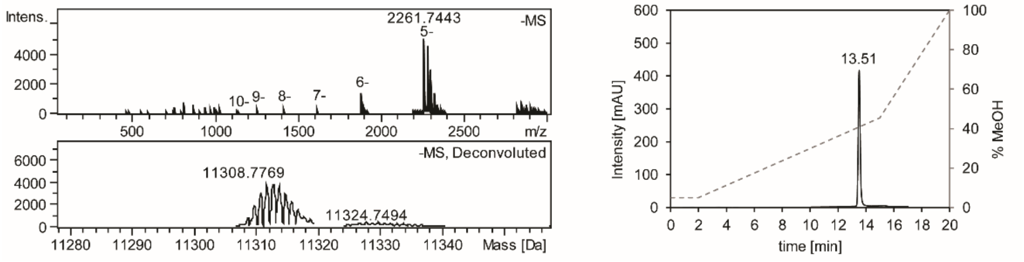

References

[1] S. Keyhani, T. Goldau, A. Blümler, A. Heckel, H. Schwalbe, *Angew. Chem. Int. Ed.* **2018**, *57*, 12017–12021; *Angew. Chem.*, **2018**, *130*, 12193-12197.

[2] I. Seven, T. Weinrich, M. Gränz, C. Grünewald, S. Brüß, I. Krstič, T. F. Prisner, A. Heckel, M. W. Göbel, *Eur. J. Org. Chem.* **2014**, *2014*, 4037–4043.

[3] J. R. Barrio, M. del C. G. Barrio, N. J. Leonard, T. E. England, O. C. Uhlenbeck, *Biochemistry* **1978**, *17*, 2077–2081.

[4] Y. V. Tarasenko, T. V. Abramova, V. I. Mamatuk, V. N. Silnikov, *Nuc. Nuc. Nuc.* **2016**, *35*, 32–42.

[5] Glen Research, *Deprotection Guide*, **2020**.
